# Incorporating farm animal models for the study of neuropsychiatric diseases: Expansion of the possibilities

**DOI:** 10.3758/s13415-025-01332-x

**Published:** 2025-08-07

**Authors:** Alexandra K. Dwulit, Rajendra A. Morey, F. Josef van der Staay

**Affiliations:** 1 Department of Molecular Biomedical Sciences, North Carolina State University College of Veterinary MedicineCVM Research Building, Room 330 A, 1051 William Moore Drive, Raleigh, NC 27607 USA; 2 Comparative Medicine Institute, North Carolina State University College of Veterinary Medicine, Raleigh, NC USA; 3 Duke-UNC Brain Imaging and Analysis Center, Duke University, Durham, NC USA; 4 Department of Population Health Sciences, Division of Farm Animal Health, Behaviour and Welfare Group, Faculty of Veterinary Medicine, University Utrecht, Utrecht, The Netherlands; 5 University Medical Center Utrecht (UMCU) Brain Center, Utrecht, The Netherlands; 6 Department of Psychiatry and Behavioral Sciences, Duke University, Durham, NC USA; 7 Mental Illness Research Education and Clinical Center for Post Deployment Mental Health, Durham VA Medical Center, Durham, NC USA

**Keywords:** Comparative neuroscience, Animal cognition, Animal welfare, Semi-experimental methods, Brain waste

## Abstract

Neuropsychiatric disorders are highly prevalent but particularly difficult to study. Animal models have been developed to understand the pathogenesis of these diseases and to develop treatments; however, the translational value of these findings has been limited. We discuss rodent and nonhuman primate (NHP) models for neuropsychiatric disorders and suggest farm animal models (FAMs) as a supplement or where NHP availability is limited and the scientific question allows it, as an alternative when feasible (i.e., when sufficient knowledge and validated methods exist). We discuss how farm animals can be relevant based on their anatomical, physiological, and brain functional similarity to humans, well-documented genetics, and complex higher cognitive functions. We discuss limitations of FAMs, including less availability of validated tools, relative lack of historical data, less expertise among livestock producers and scientists, and relatively limited understanding of functional neurocircuitry compared to rodents and NHPs. We offer guidance on the use of farm animals in research and emphasize the possibility of conducting semi-experimental studies on farms or in slaughterhouses. We argue that sustainability of research is enhanced by the availability of farm animal brains from the slaughterhouse, and generalizability is increased by using naturalistic, spontaneously occurring disease models, including from veterinary populations. The comparative validity, practicality, and ethics of using FAMs is discussed. We advocate for expanded basic science research of farm animal brains and behaviors, and conclude that FAMs may be advantageous for several reasons, although ultimately, the research question and available tools must guide the best choice of model.

**Supplementary Information:**

The online version contains supplementary material available at 10.3758/s13415-025-01332-x.

## Introduction

Neuropsychiatric diseases are prevalent, multifaceted, and complex. Although most animal studies on neuropsychiatric diseases are conducted in rodents, this approach does not always provide insights that apply to humans due to significant differences between the two species. Nonhuman primates (NHPs) frequently fill the gap between rodents and humans. We argue this gap can be bridged by complementing existing NHP research with more research on large farm animals, such as pigs, sheep, goats, and cows. When NHP availability is limited, farm animals could serve as an alternative model for particular scientific questions when they are feasible to use. The use of farm animal models (FAMs) is feasible when sufficient knowledge and validated research methods exist to answer a specific question. When sufficient knowledge is not yet available, we advocate that the knowledge gap be filled by basic research.

## Preclinical investigations on neuropsychiatric diseases

Nearly a quarter of Americans live with a mental illness (Mental Illness - National Institute of Mental Health (NIMH), 2024). Mental illnesses, especially those related to stress, are the second-leading cause of disability (Teshome et al., 2023). Unfortunately, these diseases are difficult to study for several reasons. There are no objective diagnostic tests, access to human brains with disorder-relevant cell types or structural changes is limited, neurobiology and genetics are important early in development, and there are ethical and practical concerns about studying human brains in vivo (Nestler & Hyman, 2010; Teshome et al., 2023). Alternative methods, such as in vitro, in silico, and disease modeling in human organoids, are being developed (Yao et al., 2024). However, they present significant challenges and cannot accurately reflect the physiology of a whole organism (Neziri et al., 2024).

## Most common preclinical animal models for neuropsychiatric diseases

**Fig. 1:**
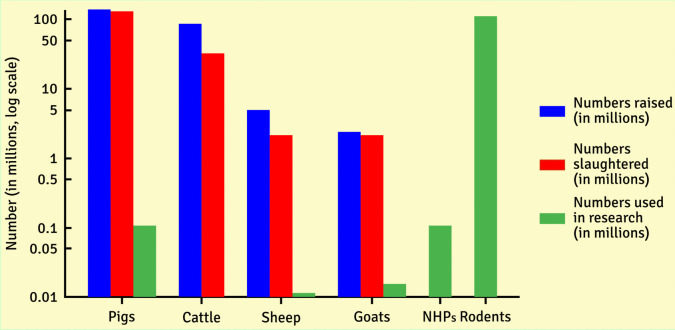
Number of brains available from slaughtered farm animals (U.S.) vs. animals used for research. The y-axis is log-scaled to better visualize the smaller numbers. Blue indicates the number of animals raised in the United States in 2023, while red indicates the number of animals slaughtered in the United States in 2023. Green indicates animals used in research in the United States in 2023 (numbers reflect those held or used in research). For cows, this number includes cattle, cows, oxen, and Watusi as the combined statistic was available. For goats, this includes only domestic goats. For rodents, the number is an estimate based on a comprehensive study performed in 2021, because the Animal Welfare Act in the United States excludes lab rats and mice, so the numbers used for research are only partially reported to government agencies. Data were obtained from multiple sources (Carbone, 2021; Cook & Schulz, 2023; *Research Facility Annual Usage Summary Report | Animal and Plant Health Inspection Service*, (Research 2023); USDA, National Agricultural Statistics Service (NASS), 2024c, 2024b, 2024a)

In neurobiological and preclinical psychiatric research, animal models are generally used to understand better the mechanisms underlying neuropsychiatric disorders and to identify and test the safety and efficacy of putative treatments (van der Staay, 2006; van der Staay et al., 2009; 2017). When developing an animal model, its reliability, replicability, and predictive, construct, and external validity must be considered (Belzung & Lemoine, 2011; van der Staay et al., 2009; Webster, 2011). Most neuroscience research is currently done on rodents or cell cultures derived from their tissues (Report from the Commission to the Council and the European Parliament Seventh Report on the Statistics on the Number of Animals Used for Experimental and Other Scientific Purposes in the Member States of the European Union, 2013). This amounts to approximately 120 million rats and mice used annually worldwide in scientific research (Cait et al., 2022), whereas a relatively low number of studies are performed on NHPs (Grimm, 2018) (Fig. 1).

### Rodent models—the workhorses of neuropsychiatric research

There are several reasons for the prevalence of rodent models: rodents are readily available, easy to manage, handle, and control in captivity, have relatively rapid maturation and reproduction, produce large numbers of offspring, and are standardized and relatively inexpensive to purchase and house (Martin et al., 2010; Walters et al., 2017). We know much about their genes, brain, cognitive abilities, and emotions relative to other species, and there exist many well-validated tests to study their emotions and cognitive abilities (Gass & Wotjak, 2013; Tanila, 2018). However, the translatability of rodent findings, especially on neuropsychiatric diseases, is limited (Keifer & Summers, 2016).

Therapeutics with proven efficacy in rodents often fail in human clinical trials (Nestler & Hyman, 2010), with available treatments for neuropsychiatric disorders effective for only ~74% of patients (Monteggia et al., 2018; Rush et al., 2006). Rodents are often housed in artificial, barren, and relatively sterile laboratory conditions where they are generally understimulated and overweight, which affects their behavior, cognitive abilities, pathophysiological changes to organs, and ultimately the translatability of research results (Martin et al., 2010; Sewell et al., 2016).

### Nonhuman primate models in neuropsychiatry

Although rodents are important in neuroscience research, it is good practice to supplement preclinical studies for drug safety and toxicology testing with research using a nonrodent species that is phylogenetically closer to humans before moving to human testing (Monticello et al., 2017; Namdari et al., 2021). Nonhuman primates, especially rhesus macaques and the common marmoset, constitute the majority of nonrodent models for neuropsychiatric disease (Scott & Bourne, 2022). Nonhuman primates have the highest construct and predictive validity for human brain disorders, because they are phylogenetically closest to humans (Phillips et al., 2014). They exhibit similar neural architecture to humans, and rhesus macaques have gyrencephalic brains like humans, although marmosets do not (Miller et al., 2016). Nonhuman primates have similar brain anatomical specialization as humans, and consequently similar functional capacity for complex behaviors (Banks et al., 2017; Eiselt & Nieder, 2013; Phillips et al., 2014). Nonhuman primates have been used to study neurodegenerative disorders, neurodevelopmental disorders, disorders that involve impairment of complex social behaviors, lifespan effects of environmental perturbation, mood and affective disorders, and substance use disorders (Banks et al., 2017; Scott & Bourne, 2022; Weerts et al., 2007).

### Large farm animal models of neuropsychiatric disease

Although rodents will remain important for studying particular questions in neuroscience, especially regarding cellular mechanisms, we argue that in addition to existing NHP models, it can be advantageous to incorporate FAMs where possible.

## Putative advantages of large FAMs

### Brain and developmental similarity with humans

**Fig. 2:**
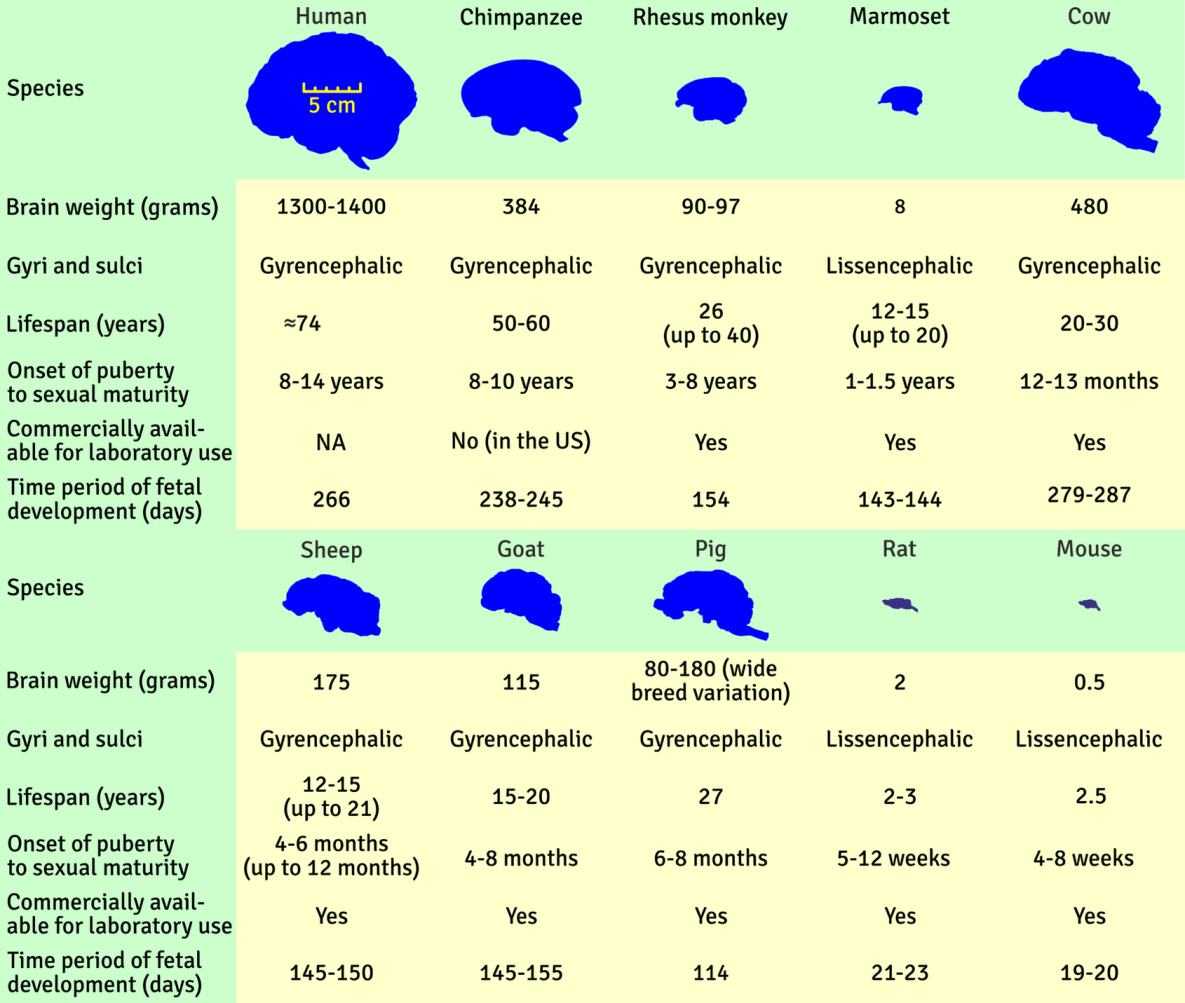
Similarities in brain structure and physiology across species. Nonhuman primates, farm animals, and rodents are compared to humans in terms of brain size, weight, anatomy, lifespan, development, and commercial availability (inspired by Figure 3.2 in van der Staay et al., 2017)

For research on neuropsychiatric diseases, it is important that the brain of the model animal is similar to that of humans. Pigs, sheep, goats, and cows all have gyrencephalic brains, such as humans (Fig. 2) (Cozzi et al., 2020; D’Souza et al., 2021; McBride & Morton, 2018; Scott & Bourne, 2022). The brains of pigs and sheep also have more human-like ratios and distributions of white and grey matter as well as regional distribution of neurotransmitter systems (Hillerer & Gimsa, 2024; Vink, 2018).

The brain structure of farm animals is similar to that of NHPs and humans. The prefrontal cortex in Göttingen minipigs and subcortex in pigs and sheep, for example, resembles that of humans and NHPs (Jelsing et al., 2006; Morton & Howland, 2013). The striatal part of the pig brain, like in NHPs, has distinct caudate and putamen structures divided by an internal capsule, in contrast to the single caudate-putamen complex found in rodents (Kornum & Knudsen, 2011). The globus pallidus internus and substantia nigra in sheep is similar to that of NHPs in size and functional organization (Morton & Howland, 2013). Sheep and pigs have neurons in their neocortex that are non-newly generated and are therefore considered "immature." Rodents, on the other hand, do not (La Rosa et al., 2020; Piumatti et al., 2018). These non-newly generated neurons enable significant brain plasticity that allows for complex biological processes, as are apparent in large-brained, long-living mammals, such as humans, pigs, and sheep (Piumatti et al., 2018). Similar brains can result in similar sensory abilities. For example, pigs have auditory sensitivity similar to that of NHPs (Kornum & Knudsen, 2011).

Neurogenesis also differs across species. The generation, migration, and differentiation of neurons occur over a much longer timescale in NHPs and farm animals than rodents (Hillerer & Gimsa, 2024), enabling extended periods of neural plasticity and structural maturation. In the hypothalamus, a neurogenic niche containing resident stem cells with self-renewal capacity has been observed in both farm animals and rodents (Batailler et al., 2014). Furthermore, hippocampal neurogenesis has been reported in pigs and sheep, but not in cows or goats, suggesting species-specific plasticity in farm animals. The subventricular zone (SVZ) of the lateral ventricle, a major site of neuroplasticity in mammals, is organized into distinct layers in pigs, cattle, and sheep—a pattern strikingly similar to that in humans (Low et al., 2013). These shared features point to conserved mechanisms of adult neuroplasticity among large-brained mammals and further distinguish farm animals from rodents in their translational value for neuroscience research.

Importantly, the larger brains of FAMs provide more tissue for analysis (Fig. 2). Also, imaging procedures have a higher resolution when the organs being imaged are larger. This could increase the translatability of findings (Arnfred et al., 2004; Danielsen et al., 2001; Mehra et al., 2012; Scott & Bourne, 2022). Larger brain size could also aid study of invasive neurosurgical therapies such as deep brain stimulation (DBS), and offer more surface of the brain or skull for electroencephalography (EEG) recordings (Gieling et al., 2011; Hoffe & Holahan, 2019; Morton & Howland, 2013). It also means brain regions of interest are larger and more clearly visible, allowing for more precise local injections of compounds via cannulas, lesioning of target areas, and deep brain recordings or electrophysiological stimulation (van der Staay et al., 2017). Pigs have large skull sinuses that make long-term implants technically challenging, if not impossible. Sheep, on the other hand, have a different cranial anatomy that allows for long-term brain recordings (Morton & Howland, 2013).

The ability to use neuroimaging methods, such as functional magnetic resonance imaging (fMRI) and diffusion tensor imaging (DTI), relatively easily on large farm animals is crucial for translational neuropsychiatric research, because it provides valuable information on the structure, function, and connectivity of the brain and has the potential to uncover biomarkers for disease, evaluate the efficacy of interventions, and develop personalized treatments. Although brain imaging in farm animals has only recently been developed, fMRI studies have found that pigs have functional homologues of primate cortical areas (Duhaime et al., 2006; Gizewski et al., 2007) and pig resting-state networks are homologous to those of humans (Netzley & Pelled, 2023; Simchick et al., 2019). Studies using DTI have also found that the myelination rate in the corpus callosum of pigs is similar to that in humans (Netzley & Pelled, 2023). In sheep, an MRI was used to monitor the brain in vivo, and gene therapy involving three different viral vectors was employed (Hamernik, 2019). Recently, awake sheep were successfully trained to participate in fMRI sessions without anesthesia or restraint. This shows promise for further study of higher-order ovine cognition. Until now, this type of study was only possible in humans and dogs (Pluchot et al., 2024).

The fetal development of farm animals is longer and slower than that of rodents (Fig. 2), with the growth and maturation timetable of the central nervous system of pigs especially similar to humans (Conrad et al., 2012; Sutkus et al., 2025; van der Staay et al., 2017). The pig brain matures like the human brain with myelination, biochemistry, and electrical activity (Fang et al., 2005). Pig, cattle, goat, and sheep brains have similar patterns of brain development with rapid growth during the perinatal period, similar to human brain development during late gestation and early infancy (Hillerer & Gimsa, 2024). Owing to a similar developmental trajectory, pigs are a good model for human neonates to study sex differences in brain development, neonatal diffuse brain injury, focal cortical contusion, intrauterine asphyxia, and hypoxia-ischemia (Kinder et al., 2019; Strawn & Behura, 2022). The time period of cow fetal development is much closer to that of humans than rhesus macaques or marmosets (Fig. 2), making cows a potentially important model for studying neurodevelopmental conditions, granted all the tools needed to study cows are available. This similar developmental trajectory is highly relevant when studying disorders such as anxiety, for which early development and neural wiring are important factors in determining future disease progression (Thapar & Riglin, 2020).

### Anatomical, functional, and physiological similarity to humans

Pigs have greater anatomical (body and organ size, cardiovascular system, skin, urinary system), functional (gastrointestinal and immune system), physiological (hematology), and metabolic similarities to humans than rodents (Hamernik, 2019), including early life physiology, diet, and gastrointestinal function. Consequently, they can be used to model various diseases and more accurately evaluate pharmacokinetics and treatment responses (Netzley & Pelled, 2023). Pigs sustain several cellular pathologies like humans and have been used to model diffuse brain injury, fluid percussion brain injury, focal scaled cortical contusion, focal cerebral contusion using a piston, acute subdural hematoma, and global brain ischemia (Dolezalova et al., 2014; Hoffe & Holahan, 2019; Kornum & Knudsen, 2011). Pigs are also commonly used to study traumatic brain injury (TBI), induced by controlled cortical impact, rotational acceleration, and blast injury (Netzley & Pelled, 2023). Bovids have been used as models for repetitive mild TBI, which can progress into chronic traumatic encephalopathy (Ackermans et al., 2021). There are also examples of farm animal models for stroke, circulatory disturbances, seizures, neuronal resilience, and restorative brain function (Vrselja et al., 2019).

While the mouse immune system is only 10% similar to the human immune system, the pig immune system is 80% similar (Schook et al., 2005). This is especially important when studying neuroimmune interactions. Owing to the similarity of platelet fatty acids in depressed humans and stressed pigs, pigs have been found to be a good animal model of depression (Cocchi et al., 2009; van der Staay, Schuurman, et al., 2010a, 2010b). Because the pharmacological homology between minipigs and NHPs is greater than for rodents based on studies with psychoactive drugs in pigs (Peacock et al., 1999; Peacock & Gerlach, 1999; van der Staay et al., 2009), pigs have also been used as models for schizophrenia, anxiety, and abnormal behavior, such as aggression and stress-related behavior (Lind et al., 2007) (Table S1).

Sheep and cows have similar cellular and molecular mechanisms to humans, making sheep a good model for Huntington’s disease and tauopathies (Ackermans et al., 2021; Morton, 2018) and cows for prion diseases, also known as transmissible spongiform encephalopathies (Asher & Gregori, 2018) (Table S1). Both sheep and goats show increasing neurofibrillary tangles and tau deposition with age, so more emphasis has been placed on studying aging in sheep and goats (Ackermans et al., 2021). Sheep also have human-like neurological symptoms in response to poisoning with neurotoxic compounds, and they have neurological diseases with pathologies and mutations similar to those in humans, including those in Batten’s disease and Gaucher’s disease (Morton & Howland, 2013).

Farm animals have a larger body size than rodents, making it easier to use clinically available equipment and techniques used on humans. For example, it is easier to collect larger volumes of blood samples more frequently on farm animals without significant changes in blood chemistry; it also enables more frequent tissue biopsies, making it is easier to study changes in hormones, metabolites, immune factors, and cellular components within the same individual (Hamernik, 2019). If parts of an animal are designated as separate experimental units (i.e., dermatological study skin patches), or when tissue samples of small brain structures must be experimentally manipulated, the sampling can be more precise and the samples larger (Gieling et al., 2011; van der Staay et al., 2017). Also, owing to their large body size, farm animals can serve as models for developing new equipment and testing the diagnostic and prognostic potential of new methods and therapies (Netzley & Pelled, 2023). For example, subdurally implanted EEG monitoring systems have been tested in pigs to assess long-term posttraumatic epilepsy (Martinez-Ramirez et al., 2022). Pigs were used to test a neural implant to improve cognitive function with the ultimate goal of helping humans with neurodegenerative diseases or spinal cord injuries (*Neuralink*, 2020).

### Genetic similarity to humans

Importantly, the genome sequence of humans is more similar to cattle and pigs than rodents (Humphray et al., 2007; Jagadesan et al., 2023; Tellam et al., 2009; Walters et al., 2017; Wernersson et al., 2005). For example, phylogenetically, pigs are threefold closer to humans than mice at the nucleotide level (Walters et al., 2017). This is especially important when studying the genetic basis of neuropsychiatric diseases. Genetically modifying farm animals through gene targeting, gene editing, and transgenesis is more feasible than modifying traditional lab animal species (Hamernik, 2019; Polejaeva et al., 2016).

### Complex cognitive functions and behaviors

Recently, there has been more emphasis on addressing the knowledge gap in farm animal cognition (Nawroth et al., 2019a, 2019b). Studying cognition and behavior is essential to understanding brain function and modeling neurobehavioral and psychiatric disorders. These functions are often impaired in people with these disorders (Netzley & Pelled, 2023). By measuring behavior and cognition in animal models, researchers can gain a greater understanding of their brain functions (sensory perception, motor control, learning and memory), social behavior, and emotional processes, which simultaneously benefits farm animal welfare and translational research. Farm animals display high levels of cognitive complexity on cognitive tests developed for various farm animals, including cattle, horses, pigs, and smaller ruminants, such as goats and sheep (Gieling et al., 2011; Nawroth et al., 2019a, 2019b; Nordquist, 2021), which are relevant to animal models of neuropsychiatric disease (Shettleworth, 2010).

## Limitations of large FAMs

Although farm animals show promise as translational models in neuroscience, there are still relatively little historical data available on farm animal brains, specifically the anatomy and connections associated with key brain areas, such as the prefrontal cortex and limbic regions, as well as information on functional neurocircuitry and neurochemistry. There is also less information on conscious cognitive processes, emotions, and sensory capabilities (Cozzi et al., 2020) relative to rodents and NHPs. Consequently, more basic research on farm animals is critical to fully appreciate their suitability for neuroscience research. Such basic research would largely depend on external funding, which can be challenging as funding opportunities are sparse. This problem was already identified by Roberts et al. (2009) a decade and half ago, yet it remains largely unresolved, and funding farm animal research on biomedical sciences has not yet gained priority, even though the economic value of farm animals often offsets much of their associated research expenses.

There is currently a lack of validated models, tests, test equipment, and genetic tools used in farm animals compared to rodents (Netzley & Pelled, 2023). Currently, validated comprehensive ethograms are missing for pigs and other farm animal species as well as valid measures for detecting when adaptive stress turns into distress (Holden, 2000; Nordquist et al., 2017). These limitations also affect NHP models. Recent advances in the manipulation of neural circuits to observe behavior and the measurement of changes in neural activity in real time, as well as genetic manipulation techniques, have mostly been established in rodents (Scott & Bourne, 2022).

There is a relative lack of expertise among animal husbandry workers and scientists about working with farm animals relative to rodents, because most neuroscience research and education is rodent-centered (Libby, 2015). This problem also exists for experimental studies using NHPs, because most NHP research is done by a small group of experts with specialized knowledge of primates.

We also know that large animals can grow fast. For example, the daily weight gain of pigs in the grower-finisher period is ~800 to 1,000 g (Pardo et al., 2013), and the brain volume of pigs doubles from 2 to 24 weeks of age (Conrad et al., 2012). The high body mass of adult pigs can potentially make them more difficult to handle, administer drugs, or test behavior (van der Staay et al., 2017). Their size could also mean they require greater quantities of drugs (van der Staay et al., 2017), which could be a limitation if only limited quantities of putative therapeutics are available. In some cases, this limitation could be mitigated by locally injecting putative therapeutics into target brain areas. Miniature versions of farm animals, such as Göttingen minipigs or Dwarf goats, circumvent some of the above limitations.

Nonhuman primate use is heavily regulated by the U.S. federal government (Feng et al., 2020; Harding, 2017), although farm animals used as biomedical models also require oversight. This includes annual USDA inspections, regulatory oversight by USDA’s Animal and Plant Health Inspection Service (APHIS), existence of Institutional Animal Care and Use Committees (IACUC), and the Animal Welfare Act (AWA), which sets minimum standards for care and treatment of regulated animals (*Research Using Agricultural Animal Species | OLAW*, n.d.). This can increase costs of using farm animals in a lab compared with a farm setting, which is an important consideration as biomedical research will mostly be done in labs. However, housing a farm animal, even a large one, is still less expensive than housing a NHP in a lab setting. This is partly because NHPs are subject to more regulations than farm animals (“Cost Analysis and Rate Setting Manual,” 2025; Phillips et al., 2014). Costs do not have to be prohibitive for farm animals, as seen in sheep models for Huntington’s disease and transmissible spongiform encephalopathies that are housed outdoors and are relatively easy to care for and maintain (Hagenaars et al., 2010; Morton & Howland, 2013).

There is currently limited commercial availability of research supplies designed for large farm animal species, as well as a lack of facilities for housing and testing animals other than rodents in most research institutes, especially if they are in urban centers (Netzley & Pelled, 2023; van der Staay et al., 2017). Sheep, goats, and pigs are social animals that live in groups, so there is a risk of inducing stress when animals are separated from their group (Arndt et al., 2009; Estevez et al., 2007). Keeping animals in their social groups requires extensive habituation to the housing conditions, experimenters, and testing environment and procedures (Antonides et al., 2015), which all takes time. If the experimental unit is the pen, barn, or farm, then experiments may be logistically challenging due to space and cost requirements (see Figure 3.6 in van der Staay et al., 2017). Fortunately, if the experiment calls for measuring multiple variables and testing different hypotheses within one study, the cost and space demands can decrease (Gieling et al., 2011).

The life expectancy of large animal model species is not well documented, although it is much higher than in rodents (Mitchell et al., 2015; Morton & Howland, 2013) (Fig. 2). This can make it difficult to study aging in farm animals compared with rodents, because the progression of neurodegenerative diseases in rodents occurs more rapidly and can be studied over a shorter time period. Conversely, this can also provide benefits; the longer lifespan of farm animals makes it possible to study neurodegenerative processes and late-stage consequences of gene therapy in a more temporally precise manner that better matches the human timescale (Ackermans, 2023; Casal & Haskins, 2006; Morton & Howland, 2013). The larger size and relatively long generation intervals makes it possible to elucidate the mechanisms by which the environment (i.e., diet, temperature, and toxicants) influences the developing embryo and fetus, resulting in adult onset of disease (Roberts et al., 2009). This is not possible in animals sent for slaughter as young adults (i.e., pigs that are kept until reaching their slaughter weight, 8–12 months of age) (Nordquist et al., 2017).

Another consideration with FAMs is greater difficulty in brain extraction from relatively thicker skulls than rodents. This is especially the case when extracting brains from slaughterhouses in a way that is useful for scientific work (i.e., no damage to the brain from stunning, rapid dissection of the brain and immediate storage). These additional considerations were taken into account in van der Staay et al. (2010a, 2010b) when trying to develop an animal model of major depressive disorder via repeated, long-term stress. The altered slaughter process that does not destroy tissue structure is more time consuming and incompatible with normal abattoir processing, bringing challenges to using animals from slaughterhouses into experimentation and might require extra investments to compensate the slaughterhouse.

## Implementation of large FAMs

While replacing farm animals in traditional lab settings may be unrealistic, supplementing traditional experiments with research in semi-experimental settings, for example in the abattoir as done in van der Staay et al. (2010a, 2010b), may prove a valuable endeavor to increase generalizability of studies. These settings provide an almost inexhaustible source of biomaterial (i.e., brains) and much larger sample sizes than would be possible in lab settings. In animal studies, a balance between generalizability and standardization must be met, and that balance can vary depending on the specific research question (van der Staay, Arndt, et al., 2010a, 2010b). When testing physiological mechanisms or when expected effect sizes are small (i.e., toxicological studies, drug safety testing), the balance should generally favor standardization. However, when trying to extrapolate and translate results from animal studies to humans, diverse mammalian models and more naturalistic settings (i.e., semi-experimental settings) can increase generalizability (Roberts et al., 2009; van der Staay et al., 2017). As long as an adequate research question is formulated and animals can differ in quantifiably relevant characteristics, such semi-experimental studies are possible and investigators should consider this option.

The availability of veterinary medical records for farm animal patients is recently increasing, because there is more popularity for miniature farm animal pet varieties (Mini Farm Animals Are Adorable. There’s Also a Growing Demand for Them, 2024). An important benefit of farm animals is the availability of veterinary medical records of large patients that have spontaneously occurring disease (Edwards et al., 2018). Greater integration between the veterinary and human biomedical community will be required to make use of such databases. For example, it could be possible to establish a network of veterinarians who report cases of farm animals with conspicuous behavior indicating neurobiological disorders to a contact person in the Departments of Psychiatry or Neurology. Brains could then be donated, dissected, and fixed according to a defined protocol via the contact person. This would be most feasible at universities that have both a veterinary and human medical faculty who are already engaged in “one health” research. If veterinary populations continue to grow, citizen science initiatives, such as the Dog Aging Project in dogs (Kaeberlein et al., 2016), could be developed for farm animals. Such initiatives could include pet farm animals, but also farm animals kept for agricultural purposes, as long as owners are willing to participate.

Miniature farm animals, such as Göttingen minipigs, are increasingly used in research. These smaller farm animals can circumvent challenges with housing and maintaining large farm animals (Mini Farm Animals Are Adorable. There’s Also a Growing Demand for Them, 2024). Often their housing prices are much cheaper as they require less space and provisioning than the larger varieties (van der Staay et al., 2017). However, they may have health issues from inbreeding. While concerning from a welfare standpoint, it may be beneficial from an opportunistic research standpoint. If these miniature animals do not show “normal” development or behavior, they can serve as spontaneous neuropsychiatric disease models.

Anxiety or related behavioral disorders could also be investigated by establishing a system, such as the *Animal donor codicil*. Such a codicil has already been introduced at Utrecht University (Animal Welfare Body Utrecht, 2016), where donated cadavers of dogs and cats are primarily used for educational purposes. This system may be extended to companion and farm animals under treatment for neuropsychiatric disorders, as their postmortem examinations could be used in scientific research projects. Such a system may best be established in cooperation with veterinarians at veterinary faculty hospitals or larger nonacademic veterinary practices staffed by research-minded veterinarians.

Early life experiences in farm animals also present opportunities for studying long-term effects of adversity (Lucas et al., 2024). Management conditions can be manipulated early in life, given approval by an ethics committee, to observe long-term effects of pre-, peri-, and early postnatal adverse events on later-life functioning. Most farm animal species, such as pigs, have more offspring per litter than NHPs (McBride & Monson, 2024), so it may be easier to test effects of different environmental conditions on siblings. Such experiments can even be integrated into existing studies with a main question that requires manipulation of feeding and housing conditions, to explore effects on neurochemistry, neuroanatomy, or gut microbiome and how this relates to cognition and behavior.

Importantly, some farm species provide natural models for diseases that are difficult to induce. Bovids provide a great opportunity for studying TBI, because these species naturally headbutt (Ackermans, 2023) and consequently may have better face, construct, and predictive validity. These species include bighorn sheep, which are not traditionally considered farm animals, but could nonetheless be tracked in the wild for headbutting behavior and postmortem examinations could be completed if samples are possible to acquire. Although males are slightly better protected by their thick skull and horns, both sexes develop TBI-related neuropathology (i.e., tauopathies) (Ackermans et al., 2022). Because it is ethically and technically difficult to induce TBI with high face validity in laboratory animals (Xiong et al., 2013), studies of farm animals may provide a solution. However, this requires a trained person, such as a caretaker or veterinarian, to notice the symptoms of TBI, although they need not be constantly present as behavior could be video recorded.

**Fig. 3:**
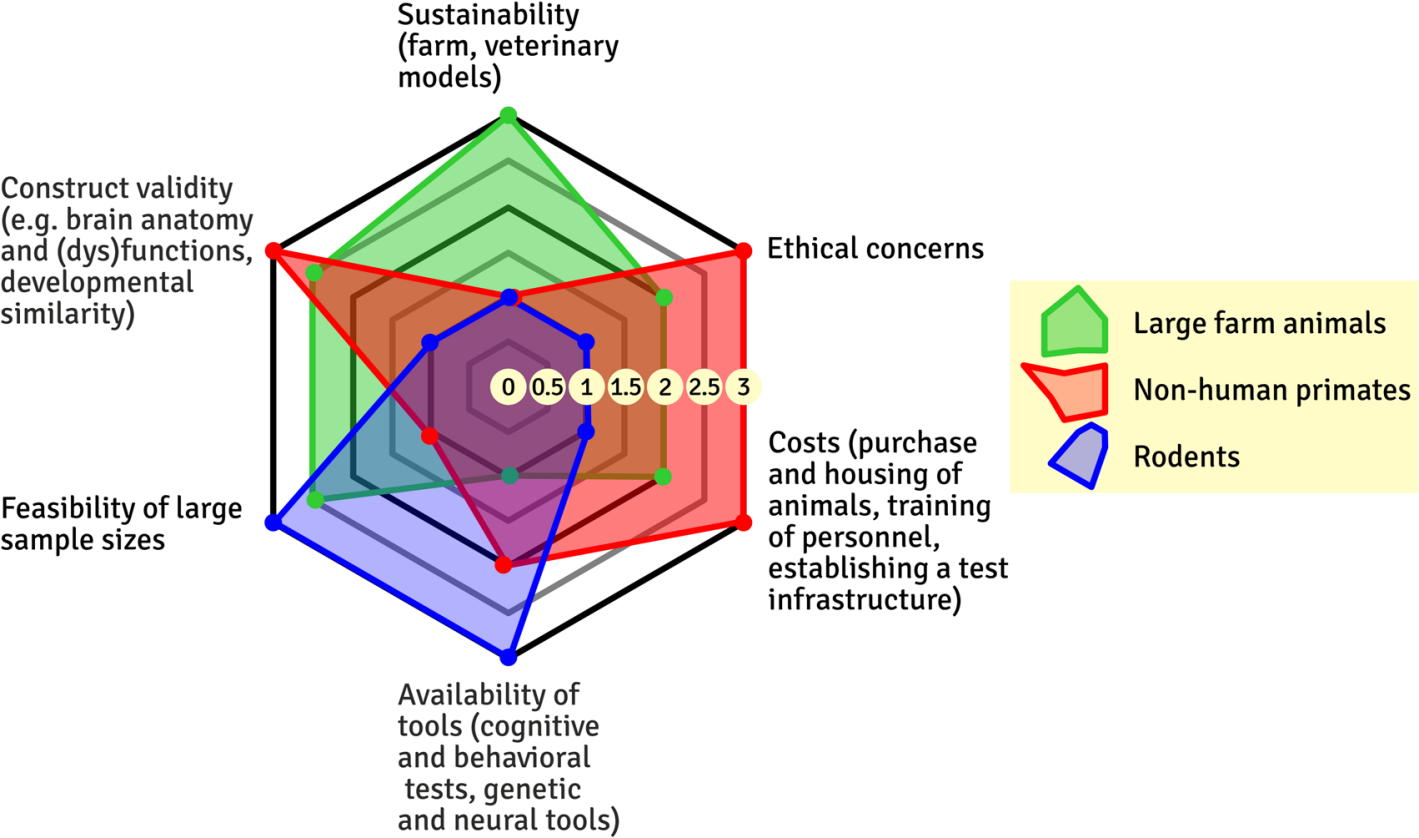
Spider plot of the hypothesized differences between studies using rodents, nonhuman primates, and large farm animals across six criteria. We rated the position of each of these three categories of animals on six criteria on a scale of 0 to 3, where 0 = absent, 1 = low, 2 = medium, and 3 = high

Figure 3 summarizes a comparison of rodents, NHPs, and large FAMs on aspects of sustainability, ethical concerns, costs, feasibility of large sample sizes, and validity.

## Discussion

### Comparative validity of large FAMs

Progress in developing animal models of neuropsychiatric disease has been frustratingly slow, partly due to the difficulty of modeling such disorders in animals, but also due to the overreliance on rodent models. Although a large animal model is needed after rodent testing, NHPs cannot always fill this gap. We advocate expanding the use of FAMs to complement existing large animal research. When NHPs are unavailable, using FAMs for particular questions may be a feasible alternative, provided sufficient relevant knowledge and validated research methods exist. We emphasize that expanding the diversity of animal models by including farm animals, where feasible and appropriate, can broaden generalizability and richness of neuroscience findings. Similar findings across various nonhuman species increases the likelihood that the findings will be translationally relevant to human outcomes.

The prevailing viewpoint is that NHPs can provide better construct validity than most other animal models, because they are phylogenetically closer to humans. This is not always the case, depending on what is being investigated. When considering what we already know about farm animal neurodevelopment, brain homology, or physiology, farm animals may be more similar to humans than NHPs, such as marmosets or even rhesus macaques. In practice, however, we know much more about the functional neurocircuitry and neurochemistry of NHPs than we do about the brains of farm animals. Until we conduct more basic research on farm animals, it may be challenging to know exactly to what extent they can complement, or even be alternatives, to other models. It is thus imperative that we expand basic science efforts to develop additional models for neuropsychiatric disease that can broaden the generalizability of neuroscience findings.

A particular advantage of using farm animal models is the ability to study them in seminaturalistic settings and the consequent ecological validity and generalizability that comes from such studies, which is unprecedented in traditional lab animal models. Farm animals can be studied in laboratory settings, farm settings, slaughterhouses, or as veterinary patients in the clinic. With comparative construct validity to NHPs, farm animal models enable animal modeling to be done through the lens of sustainability, ecological relevance, and species-specific strengths, offering a pathway toward more generalizable and ethically robust translational neuroscience.

### Comparative practical considerations of large FAMs

The validity of FAMs may match NHPs in some areas, but practical considerations are also very important to consider when choosing animal models. There are fewer validated cognitive and behavioral tests for farm animals than rodents or NHPs. Genetic and neural tools lag behind rodents in both farm animals and NHPs (Scott & Bourne, 2022). There is a dire need for validated methods in farm animals. Research areas of priority for FAMs would include areas for which validated methods already exist, such as investigating environmental and development factors of disease.

Importantly, methods for measuring emotions, motivation, cognitive abilities, brains, and genetics are being actively developed and used in the farm animal welfare community (Tables S1 and S2). These include high resolution, 3D stereotaxic brain atlases, behavioral and cognitive tests, techniques to manipulate genes, and neuropsychiatric disease models (Klymiuk et al., 2012; Mendl & Paul, 2020; Murphy et al., 2014; Nawroth & Langbein, 2019; Roelofs et al., 2016). Automated tests of high throughput cognitive function, similar to those in NHPs, have been developed for medium-sized quadrupeds, including sheep and pigs, and can be used in farm animal settings (McBride et al., 2016). Unfortunately, such developments often go unnoticed by neuroscientists. For example, many journals devoted to farm animal welfare research are not included in major biomedical databases, limiting exposure of biomedical scientists to relevant literature on farm animals. It will be important moving forward to emphasize collaborative efforts between animal welfare and neuroscience communities to address this gap.

Nonhuman primate models can be standardized in lab environments, although owing to small sample sizes, diversity of species used, and heterogeneity of behavioral experiments, standardization of NHPs can be limited (Scott & Bourne, 2022). A major limitation in neuroscience, especially in cognitive neuroscience and psychology, is the low statistical power of studies owing to small sample sizes (Barroca et al., 2022). If the current number of animals is insufficient to reach robust conclusions, animals will be unnecessarily used or sacrificed in underpowered experiments (Sena et al., 2010; van der Staay et al., 2017). Studies needing larger sample sizes can make use of farm animals (including their miniature varieties), because there is almost an unlimited variety of farm animals of different breeds from different suppliers that can be used in laboratory conditions or settings outside the laboratory (Nordquist et al., 2017; Renggaman et al., 2015; Simianer & Köhn, 2010; van der Staay et al., 2017).

There are vast numbers of domestic livestock slaughtered for food that could potentially be involved in experimental studies or opportunistic sampling. For example, in 2023, approximately 128 million hogs, 32.8 million cattle, and 2.17 million sheep were slaughtered in the United States (USDA, National Agricultural Statistics Service (NASS), 2024b) (Fig. 1). This can provide a significant number of animals whose brains can be incorporated into research. Farm animal brains can be used directly after slaughter, or neural tissue can be sampled from subjects in slaughterhouses under closely monitored conditions (Cozzi et al., 2020).

For optimal generalizability and translatability, both standardized experiments as well as consideration of natural group heterogeneity are important (van der Staay, Arndt, et al., 2010a, 2010b). When focusing on research where standardization is key, farm animal breeds are helpful. There are strains of dairy cows and pigs that have very homogeneous genetic backgrounds matching that of lab animals (Cozzi et al., 2020). Conversely, for research questions emphasizing generalizability, several crossbred lines are available, such as pigs used for meat production whose parent lines are not inbred. There is a nearly unrestricted availability of outbred species and strains from controlled suppliers (i.e., miniature pigs and commercial pig breeds) with known and controlled health status that can be used for research (van der Staay et al., 2017).

There are strict regulations for use of NHPs in biomedical research. Although this is also the case for farm animals, the regulations are slightly less burdensome. The costs of studying farm animals in biomedical settings, even with USDA oversight, are less than NHPs; the costs of farm animals in farm settings, where USDA oversight is not required, are even less (“Cost Analysis and Rate Setting Manual,” 2025). The animal model that is most adequate for answering a scientific question should be preferred; however, in addition to scientific criteria, such as reliability, validity, generalizability, and relevance of the animal model used, practical aspects, such as the availability, affordability of the study, and available infrastructure (housing, testing capacity, trained personnel), are crucial (van der Staay et al., 2009, 2017).

**Fig. 4:**
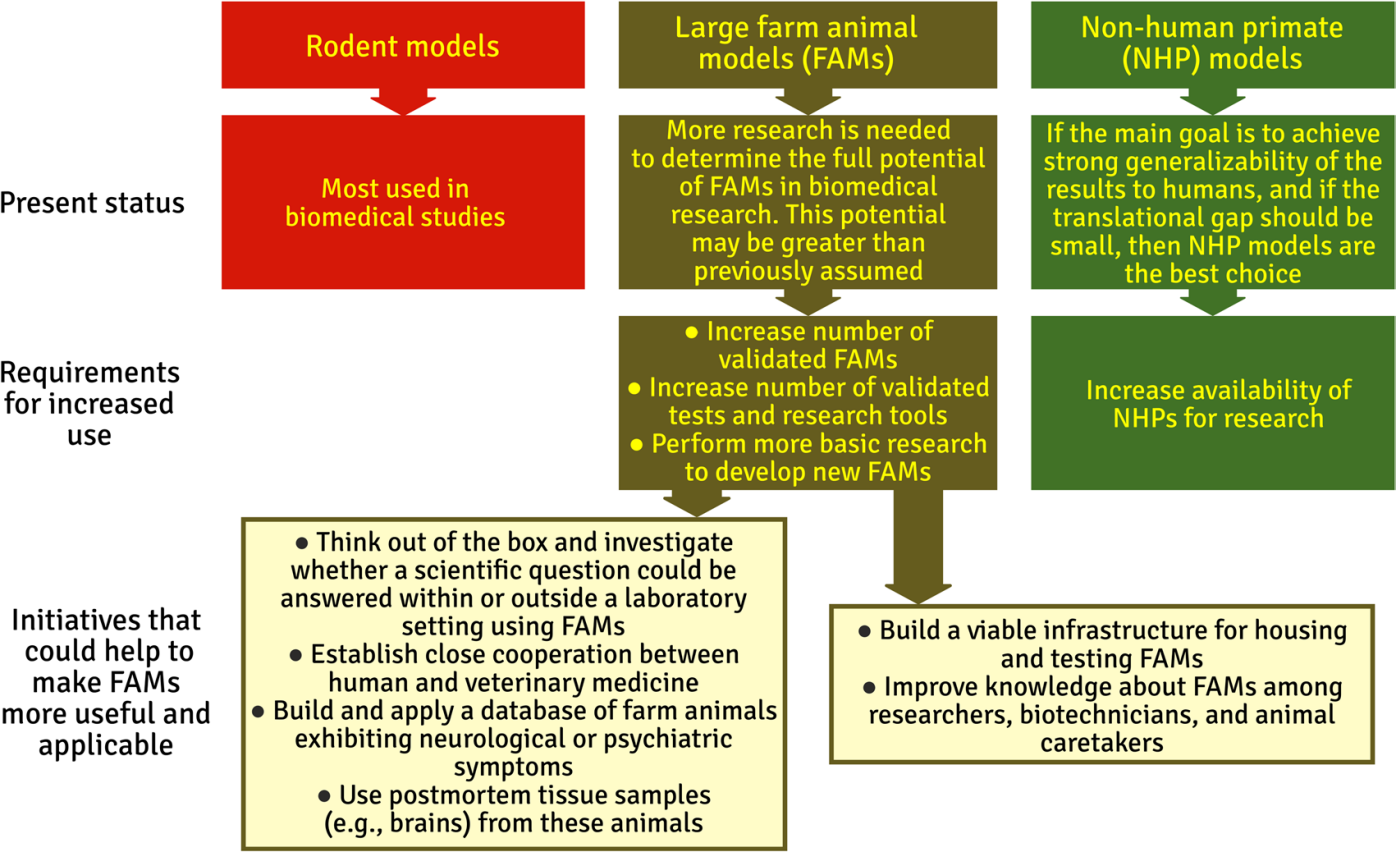
Summary of a comparison between rodent, farm animal, and nonhuman primate models. Included is the present status of these models, requirements for increased use of farm animal and nonhuman primate models, and initiatives that could make farm animal models more useful and applicable

Nonhuman primates and rodents have been used in neuroscience research for decades, sometimes for reasons that are not scientific (e.g., the laboratory infrastructure and staff expertise is rodent-centric). Where alternative models are more appropriate, reasons for adhering to traditional models include familiarity of the researchers, availability of the model, lack of FAM housing and testing facilities, lack of resources, cost, and bias in neglecting publications that use alternative models (Prior et al., 2021; van der Staay et al., 2017). Alternative large animals are often not considered, which could be a reason why FAM use is still in its infancy and the potential of these models cannot fully be estimated. We anticipate that as more groups consider the use of FAMs, more ideas will be generated on how to implement them in practice (see Fig. 4 for a summary overview).

### Comparative ethical considerations of large FAMs

Scientists often prioritize practical reasons (ease of care, space for animal maintenance, reproduction rates, and cost) when choosing an animal species for neurobiological studies (Żakowski, 2020). Farm animals share many practical considerations with NHPs, as previously discussed. However, the use of NHPs is facing strong public opposition and ethical concerns (Bailey, 2005; Schroeder et al., 2018). Compared with NHPs, farm animals have a smaller perceived similarity with humans and a larger empathic distance, with high anatomical, physiological, and genetic similarity to humans (Gieling et al., 2011). Research with FAMs involving sacrificing the animal presents a lower ethical concern considering the FAM is generally destined for slaughter. In a study conducted on individuals from various parts of Spain, phylogenetic distance was an important factor in determining ethics of a particular model, with more support for pigs than monkeys in biomedical research (Goñi-Balentziaga et al., 2022). However, the issue of public support for NHPs versus farm animals in research is complicated, because this relates to many factors, including type of research done, cultural factors, and familiarity with species (Ormandy & Schuppli, 2014).

Research on farm animal brains, neuroanatomy, and neurophysiology that underlies their behavior could lead to improvements in farm animal welfare and an improved understanding of FAM brain use for neuroscience research. Farm animals have the possibility of being studied in the farm setting, an environment that they have been selected to live in for thousands of years. When studying animals in a farm setting, it could be possible to return farm animals to the food chain for human consumption if experiments did not involve treatment with compounds that would harm the consumer or that are not yet approved for human use, although often the waiting time for drug clearance is not insignificant (van der Staay et al., 2017).

The organs and brains of farm animals are available in millions (Cozzi et al., 2020), and as the world is moving toward reducing waste and recycling materials, we need more sustainable solutions for research. This multipurpose approach follows the principle of the 3Rs (replace, reduce, refine; see Grimm et al., 2023; Russell & Birch, 1959/1992), which is legally required in many countries including the United States, European Union member states, and other countries with strict animal welfare regulations (Nordquist et al., 2017). It is also the dominant ethical framework in animal research policy (Grimm et al., 2023).

The sustainable use of bodies and organs in biomedical research has also been implemented for other species. For example, deceased companion animals (mainly dogs and cats) can be donated by their owners and may be used in research through the animal donor codicil in the Netherlands (Animal Welfare Body Utrecht, 2016). While we do not argue that farm animals on farms would replace those housed in facilities that require USDA inspection, control, and authority, we argue that such initiatives would provide additional options when more naturalistic and less standardized settings would help to answer particular questions.

It has been recognized that the use of rodents and NHPs for research in seminatural environments can improve welfare and scientific validity (Hernández-Arteaga & Ågmo, 2023). Regardless, such approaches remain underutilized as their feasibility and potential have not yet been systematically investigated. Increasingly, using animals that display similar diseases to humans, such as anxiety-like behavior, spontaneously and in naturalistic settings, may improve generalizability within a species as well as in contexts outside of standardized conditions, especially with the advent of automated behavioral analysis (Devinsky et al., 2018; Puścian & Knapska, 2022). It has also been suggested that the use of animal models in seminatural environments could prove revolutionary for translational psychiatry (Shemesh & Chen, 2023). This type of work not only improves the welfare of species studied compared with traditional lab settings (Makowska & Weary, 2016) but can improve generalizability and is especially important when studying complex behavioral phenomena like those in neuropsychiatric diseases (Hernández-Arteaga & Ågmo, 2023). It has been argued that variable environments provide results with greater predictive validity and replicability (Hernández-Arteaga & Ågmo, 2023), and should be implemented where possible.

We have domesticated and created distinct breeds of farm animals through generations of selective breeding, and although their management conditions are often not fully naturalistic, domesticated species may be more comfortable with human interaction than wild or laboratory animals. Even captive-bred wild species still exhibit differences from their domesticated counterparts (Künzl et al., 2003). Human stressors—such as urban density or social instability—may also be more analogous to those experienced by free-ranging or farmed animals than by laboratory models. Accounting for animal agency and allowing for natural behavior can provide more reliable insights for biomedical research to understand the relationship between brain and behavior (Sueur et al., 2011). Provided that the available tools are adequate and the research questions can be addressed by using large FAMs, such research could complement traditional laboratory studies.

Current research practices can be enriched by using farm animals as appropriate models in either laboratory or seminaturalistic settings. By tapping into already-available animals (i.e., postagricultural use or slaughterhouses), we can reduce the number of animals bred for research. Importantly, where appropriate and feasible, using farm animals as opposed to NHPs may quell some public concern. Replacing in vivo animal testing with in vitro, in silico, or organoid approaches would also be a highly desirable goal, and there are numerous initiatives to this end. At present, in vivo animal experiments are still indispensable for answering scientific questions, particularly those investigating the whole organism and its interactions with external factors, and new in vivo approaches also should be considered and established in practice.

## Conclusions

The future of translational neuropsychiatry may lie not only in more refined rodent models or the use of NHPs, but in embracing underused, scalable systems like farm animals—particularly when studied under naturalistic or semi-experimental conditions. Systematic development of validated cognitive, behavioral, genetic, and neuroscience tools will be critical as will establishing collaborations between veterinarians and human mental health practitioners and researchers. Given the limited availability of NHPs and the ethical debate that limits their adoption, farm animals may be a useful addition to neuroscience research under precisely defined conditions and circumstances. Neither NHPs, FAMs, laboratory conditions, nor naturalistic conditions are appropriate for all research questions. The cost-benefit calculation will largely depend on the nature of the research questions and available infrastructure and tools. Implementing our ideas will require researchers to think outside the box and systematically inventory the scientific questions under consideration. An intensive inventory of the advantages and disadvantages of FAMs will provide a better overview of the feasibility of FAMs, especially when it comes to options for conducting research outside the laboratory (e.g., on the farm, in the slaughterhouse, or in the clinic).

## Supplementary Information

Below is the link to the electronic supplementary material.

Supplementary file1 (DOCX 124 KB)

## Data Availability

Not applicable

## Declarations

### Open practices statement

Any relevant data and materials are available upon reasonable request from the corresponding author. This study was not pre-registered.

## References

[CR1] Ackermans, N. L. (2023). Neurobiological tradeoffs of headbutting bovids. *Trends in Neurosciences,**46*(11), 898–900. 10.1016/j.tins.2023.08.004 37734961 10.1016/j.tins.2023.08.004

[CR2] Ackermans, N. L., Varghese, M., Wicinski, B., Torres, J., De Gasperi, R., Pryor, D., Elder, G. A., Gama Sosa, M. A., Reidenberg, J. S., Williams, T. M., & Hof, P. R. (2021). Unconventional animal models for traumatic brain injury and chronic traumatic encephalopathy. *Journal of Neuroscience Research,**99*(10), 2463–2477. 10.1002/jnr.24920 34255876 10.1002/jnr.24920 PMC8596618

[CR3] Ackermans, N. L., Varghese, M., Williams, T. M., Grimaldi, N., Selmanovic, E., Alipour, A., Balchandani, P., Reidenberg, J. S., & Hof, P. R. (2022). Evidence of traumatic brain injury in headbutting bovids. *Acta Neuropathologica,**144*(1), 5–26. 10.1007/s00401-022-02427-2 35579705 10.1007/s00401-022-02427-2 PMC9217783

[CR4] Animal Welfare Body Utrecht. (2016). *Animal Welfare Body Utrecht*. Retrieved November 13, 2024, from https://ivd-utrecht.nl/en/infocentre/animal-donor-codicil

[CR5] Antonides, A., Schoonderwoerd, A. C., Nordquist, R. E., & van der Staay, F. J. (2015). Very low birth weight piglets show improved cognitive performance in the spatial cognitive holeboard task. *Frontiers in Behavioral Neuroscience*. 10.3389/fnbeh.2015.00043 25774127 10.3389/fnbeh.2015.00043 PMC4343021

[CR6] Arndt, S. S., Laarakker, M. C., van Lith, H. A., van der Staay, F. J., Gieling, E., Salomons, A. R., van’t Klooster, J., & Ohl, F. (2009). Individual housing of mice—Impact on behaviour and stress responses. *Physiology & Behavior*, *97*(3), 385–393. 10.1016/j.physbeh.2009.03.008 10.1016/j.physbeh.2009.03.008 19303031

[CR7] Arnfred, S. M., Lind, N. M., Hansen, A. K., & Hemmingsen, R. P. (2004). Pre-pulse inhibition of the acoustic startle eye-blink in the Göttingen minipig. *Behavioural Brain Research,**151*(1–2), 295–301. 10.1016/j.bbr.2003.09.003 15084445 10.1016/j.bbr.2003.09.003

[CR8] Asher, D. M., & Gregori, L. (2018). Human transmissible spongiform encephalopathies: Historic view. *Handbook of Clinical Neurology,**153*, 1–17. 10.1016/B978-0-444-63945-5.00001-5 29887130 10.1016/B978-0-444-63945-5.00001-5

[CR9] Bailey, J. (2005). Non-human primates in medical research and drug development: A critical review. *Biogenic Amines,**19*(4), 235–255. 10.1163/156939105774647385

[CR10] Banks, M. L., Czoty, P. W., & Negus, S. S. (2017). Utility of nonhuman primates in substance use disorders research. *ILAR Journal,**58*(2), 202–215. 10.1093/ilar/ilx014 28531265 10.1093/ilar/ilx014 PMC5886327

[CR11] Barroca, N. C. B., Della Santa, G., Suchecki, D., García-Cairasco, N., & Umeoka, EHdeL. (2022). Challenges in the use of animal models and perspectives for a translational view of stress and psychopathologies. *Neuroscience and Biobehavioral Reviews,**140*, Article 104771. 10.1016/j.neubiorev.2022.104771 35817171 10.1016/j.neubiorev.2022.104771

[CR12] Batailler, M., Droguerre, M., Baroncini, M., Fontaine, C., Prevot, V., & Migaud, M. (2014). DCX-expressing cells in the vicinity of the hypothalamic neurogenic niche: A comparative study between mouse, sheep, and human tissues. *Journal of Comparative Neurology,**522*(8), 1966–1985. 10.1002/cne.23514 24288185 10.1002/cne.23514

[CR13] Belzung, C., & Lemoine, M. (2011). Criteria of validity for animal models of psychiatric disorders: Focus on anxiety disorders and depression. *Biology of Mood & Anxiety Disorders,**1*(1), 9. 10.1186/2045-5380-1-9 22738250 10.1186/2045-5380-1-9 PMC3384226

[CR14] Cait, J., Cait, A., Scott, R. W., Winder, C. B., & Mason, G. J. (2022). Conventional laboratory housing increases morbidity and mortality in research rodents: Results of a meta-analysis. *Bmc Biology,**20*(1), 15. 10.1186/s12915-021-01184-0 35022024 10.1186/s12915-021-01184-0 PMC8756709

[CR15] Carbone, L. (2021). Estimating mouse and rat use in American laboratories by extrapolation from Animal Welfare Act-regulated species. *Scientific Reports,**11*(1), 493. 10.1038/s41598-020-79961-0 33436799 10.1038/s41598-020-79961-0 PMC7803966

[CR16] Casal, M., & Haskins, M. (2006). Large animal models and gene therapy. *European Journal of Human Genetics,**14*(3), 266–272. 10.1038/sj.ejhg.5201535 16333317 10.1038/sj.ejhg.5201535

[CR17] Cocchi, M., Sardi, L., Tonello, L., & Martelli, G. (2009). Do mood disorders play a role in pig welfare? *Italian Journal of Animal Science,**8*(4), 691–704. 10.4081/ijas.2009.691

[CR18] Conrad, M. S., Dilger, R. N., & Johnson, R. W. (2012). Brain growth of the domestic pig (*Sus scrofa*) from 2 to 24 weeks of age: A longitudinal MRI study. *Developmental Neuroscience,**34*(4), 291. 10.1159/000339311 22777003 10.1159/000339311 PMC3646377

[CR19] Cook, H., & Schulz, L. (2023). The United States Pork Industry 2023: Current Structure and Economic Importance. *National Pork Producers Council*. chrome-extension://efaidnbmnnnibpcajpcglclefindmkaj/https://nppc.org/wp-content/uploads/2024/06/U.S.-Economic-Contribution-Report-6.28.24.pdf

[CR20] Cost Analysis and Rate Setting Manual. (2025, February 5). *Animal Care & Use Program*. https://animalcare.umich.edu/business-services/rates/

[CR21] Cozzi, B., Bonfanti, L., Canali, E., & Minero, M. (2020). Brain waste: The neglect of animal brains. *Frontiers in Neuroanatomy,**14*, Article 573934. 10.3389/fnana.2020.573934 33304245 10.3389/fnana.2020.573934 PMC7693423

[CR22] Danielsen, E. H., Smith, D. F., Andersen, F., Gee, A. D., Bender, D., Hansen, S. B., Hermansen, F., Østergaard, L., Cumming, P., & Gjedde, A. (2001). FDOPA metabolism in the adult porcine brain: Influence of tracer circulation time and VOI selection on estimates of striatal DOPA decarboxylation. *Journal of Neuroscience Methods,**111*(2), 157–168. 10.1016/s0165-0270(01)00453-8 11595282 10.1016/s0165-0270(01)00453-8

[CR23] Devinsky, O., Boesch, J. M., Cerda-Gonzalez, S., Coffey, B., Davis, K., Friedman, D., Hainline, B., Houpt, K., Lieberman, D., Perry, P., Prüss, H., Samuels, M. A., Small, G. W., Volk, H., Summerfield, A., Vite, C., Wisniewski, T., & Natterson-Horowitz, B. (2018). A cross-species approach to disorders affecting brain and behaviour. *Nature Reviews Neurology,**14*(11), 677–686. 10.1038/s41582-018-0074-z 30287906 10.1038/s41582-018-0074-z

[CR24] Dolezalova, D., Hruska-Plochan, M., Bjarkam, C. R., Sørensen, J. C. H., Cunningham, M., Weingarten, D., Ciacci, J. D., Juhas, S., Juhasova, J., Motlik, J., Hefferan, M. P., Hazel, T., Johe, K., Carromeu, C., Muotri, A., Bui, J., Strnadel, J., & Marsala, M. (2014). Pig models of neurodegenerative disorders: Utilization in cell replacement-based preclinical safety and efficacy studies. *Journal of Comparative Neurology,**522*(12), 2784–2801. 10.1002/cne.23575 24610493 10.1002/cne.23575

[CR25] D’Souza, J. F., Price, N. S. C., & Hagan, M. A. (2021). Marmosets: A promising model for probing the neural mechanisms underlying complex visual networks such as the frontal–parietal network. *Brain Structure and Function,**226*(9), 3007–3022. 10.1007/s00429-021-02367-9 34518902 10.1007/s00429-021-02367-9 PMC8541938

[CR26] Duhaime, A.-C., Saykin, A. J., McDonald, B. C., Dodge, C. P., Eskey, C. J., Darcey, T. M., Grate, L. L., & Tomashosky, P. (2006). Functional magnetic resonance imaging of the primary somatosensory cortex in piglets. *Journal of Neurosurgery,**104*(4 Suppl), 259–264. 10.3171/ped.2006.104.4.259 16619637 10.3171/ped.2006.104.4.259

[CR27] Edwards, G. L., Azain, M. J., & Parks, A. (2018). Agricultural animals as biomedical models: Occupational health and safety considerations. *Ilar Journal,**59*(2), 161–167. 10.1093/ilar/ily013 30476116 10.1093/ilar/ily013

[CR28] Eiselt, A.-K., & Nieder, A. (2013). Representation of abstract quantitative rules applied to spatial and numerical magnitudes in primate prefrontal cortex. *Journal of Neuroscience,**33*(17), 7526–7534. 10.1523/JNEUROSCI.5827-12.2013 23616557 10.1523/JNEUROSCI.5827-12.2013 PMC6619563

[CR29] Estevez, I., Andersen, I.-L., & Nævdal, E. (2007). Group size, density and social dynamics in farm animals. *Applied Animal Behaviour Science,**103*(3), 185–204. 10.1016/j.applanim.2006.05.025

[CR30] Fang, M., Lorke, D. E., Li, J., Gong, X., Yew, J. C. C., & Yew, D. T. (2005). Postnatal changes in functional activities of the pig’s brain: A combined functional magnetic resonance imaging and immunohistochemical study. *Neuro-Signals,**14*(5), 222–233. 10.1159/000088638 16301837 10.1159/000088638

[CR31] Feng, G., Jensen, F. E., Greely, H. T., Okano, H., Treue, S., Roberts, A. C., Fox, J. G., Caddick, S., Poo, M., Newsome, W. T., & Morrison, J. H. (2020). Opportunities and limitations of genetically modified nonhuman primate models for neuroscience research. *Proceedings of the National Academy of Sciences of the United States of America,**117*(39), 24022. 10.1073/pnas.2006515117 32817435 10.1073/pnas.2006515117 PMC7533691

[CR32] Gass, P., & Wotjak, C. (2013). Rodent models of psychiatric disorders—Practical considerations. *Cell and Tissue Research,**354*(1), 1–7. 10.1007/s00441-013-1706-7 23982420 10.1007/s00441-013-1706-7

[CR33] Gieling, E. T., Schuurman, T., Nordquist, R. E., & van der Staay, F. J. (2011). The pig as a model animal for studying cognition and neurobehavioral disorders. *Current Topics in Behavioral Neurosciences,**7*, 359–383. 10.1007/7854_2010_112 21287323 10.1007/7854_2010_112

[CR34] Gizewski, E. R., Schanze, T., Bolle, I., de Greiff, A., Forsting, M., & Laube, T. (2007). Visualization of the visual cortex in minipigs using fMRI. *Research in Veterinary Science,**82*(3), 281–286. 10.1016/j.rvsc.2006.08.004 17064742 10.1016/j.rvsc.2006.08.004

[CR35] Goñi-Balentziaga, O., Ortega-Saez, I., Vila, S., & Azkona, G. (2022). A survey on the use of mice, pigs, dogs and monkeys as animal models in biomedical research in Spain. *Laboratory Animal Research,**38*(1), 14. 10.1186/s42826-022-00124-5 35655241 10.1186/s42826-022-00124-5 PMC9161537

[CR36] Grimm, D. (2018). U.S. labs using a record number of monkeys. *Science,**362*(6415), 630. 10.1126/science.362.6415.630 30409868 10.1126/science.362.6415.630

[CR37] Grimm, H., Biller-Andorno, N., Buch, T., Dahlhoff, M., Davies, G., Cederroth, C. R., Maissen, O., Lukas, W., Passini, E., Törnqvist, E., Olsson, I. A. S., & Sandström, J. (2023). Advancing the 3Rs: Innovation, implementation, ethics and society. *Frontiers in Veterinary Science*. 10.3389/fvets.2023.1185706 37396988 10.3389/fvets.2023.1185706 PMC10310538

[CR38] Hagenaars, T. J., Melchior, M. B., Bossers, A., Davidse, A., Engel, B., & van Zijderveld, F. G. (2010). Scrapie prevalence in sheep of susceptible genotype is declining in a population subject to breeding for resistance. *BMC Veterinary Research,**6*(1), 25. 10.1186/1746-6148-6-25 20470415 10.1186/1746-6148-6-25 PMC2883980

[CR39] Hamernik, D. L. (2019). Farm animals are important biomedical models. *Animal Frontiers,**9*(3), 3–5. 10.1093/af/vfz026 32002256 10.1093/af/vfz026 PMC6951888

[CR40] Harding, J. D. (2017). Nonhuman primates and translational research: Progress, opportunities, and challenges. *ILAR Journal,**58*(2), 141–150. 10.1093/ilar/ilx033 29253273 10.1093/ilar/ilx033 PMC5886318

[CR41] Hernández-Arteaga, E., & Ågmo, A. (2023). Seminatural environments for rodent behavioral testing: A representative design improving animal welfare and enhancing replicability. *Frontiers in Behavioral Neuroscience,**17*, 1192213. 10.3389/fnbeh.2023.1192213 37424748 10.3389/fnbeh.2023.1192213 PMC10323197

[CR42] Hillerer, K. M., & Gimsa, U. (2024). Adult neurogenesis and the microbiota-gut-brain axis in farm animals: Underestimated and understudied parameters for improving welfare in livestock farming. *Frontiers in Neuroscience,**18*, 1493605. 10.3389/fnins.2024.1493605 39664450 10.3389/fnins.2024.1493605 PMC11631930

[CR43] Hoffe, B., & Holahan, M. R. (2019). The Use of Pigs as a Translational Model for Studying Neurodegenerative Diseases. *Frontiers in Physiology*, *10*. Scopus. 10.3389/fphys.2019.00838 10.3389/fphys.2019.00838 PMC6635594 31354509

[CR44] Holden, C. (2000). Researchers pained by effort to define distress precisely. *Science,**290*(5496), 1474–1475. 11185495 10.1126/science.290.5496.1474

[CR45] Humphray, S. J., Scott, C. E., Clark, R., Marron, B., Bender, C., Camm, N., Davis, J., Jenks, A., Noon, A., Patel, M., Sehra, H., Yang, F., Rogatcheva, M. B., Milan, D., Chardon, P., Rohrer, G., Nonneman, D., de Jong, P., Meyers, S. N., … Rogers, J. (2007). A high utility integrated map of the pig genome. *Genome Biology*, *8*(7), R139. 10.1186/gb-2007-8-7-r139 10.1186/gb-2007-8-7-r139 PMC2323232 17625002

[CR46] Jagadesan, S., Mondal, P., Carlson, M. A., & Guda, C. (2023). Evaluation of five mammalian models for human disease research using genomic and bioinformatic approaches. *Biomedicines,**11*(8), 2197. 10.3390/biomedicines11082197 37626695 10.3390/biomedicines11082197 PMC10452283

[CR47] Jelsing, J., Nielsen, R., Olsen, A. K., Grand, N., Hemmingsen, R., & Pakkenberg, B. (2006). The postnatal development of neocortical neurons and glial cells in the Göttingen minipig and the domestic pig brain. *Journal of Experimental Biology,**209*(8), 1454–1462. 10.1242/jeb.02141 16574805 10.1242/jeb.02141

[CR48] Kaeberlein, M., Creevy, K. E., & Promislow, D. E. L. (2016). The dog aging project: Translational geroscience in companion animals. *Mammalian Genome,**27*(7–8), 279–288. 10.1007/s00335-016-9638-7 27143112 10.1007/s00335-016-9638-7 PMC4936929

[CR49] Keifer, J., & Summers, C. H. (2016). Putting the “Biology” Back into “Neurobiology”: The Strength of Diversity in Animal Model Systems for Neuroscience Research. *Frontiers in Systems Neuroscience*, *10*. 10.3389/fnsys.2016.00069 10.3389/fnsys.2016.00069 PMC4992696 27597819

[CR50] Kinder, H. A., Baker, E. W., & West, F. D. (2019). The pig as a preclinical traumatic brain injury model: Current models, functional outcome measures, and translational detection strategies. *Neural Regeneration Research,**14*(3), 413–424. 10.4103/1673-5374.245334 30539807 10.4103/1673-5374.245334 PMC6334610

[CR51] Klymiuk, N., Böcker, W., Schönitzer, V., Bähr, A., Radic, T., Fröhlich, T., Wünsch, A., Keßler, B., Kurome, M., Schilling, E., Herbach, N., Wanke, R., Nagashima, H., Mutschler, W., Arnold, G. J., Schwinzer, R., Schieker, M., & Wolf, E. (2012). First inducible transgene expression in porcine large animal models. *FASEB Journal,**26*(3), 1086–1099. 10.1096/fj.11-185041 22138035 10.1096/fj.11-185041

[CR52] Kornum, B. R., & Knudsen, G. M. (2011). Cognitive testing of pigs (*Sus scrofa*) in translational biobehavioral research. *Neuroscience and Biobehavioral Reviews,**35*(3), 437–451. 10.1016/j.neubiorev.2010.05.004 20553757 10.1016/j.neubiorev.2010.05.004

[CR53] Künzl, C., Kaiser, S., Meier, E., & Sachser, N. (2003). Is a wild mammal kept and reared in captivity still a wild animal? *Hormones and Behavior,**43*(1), 187–196. 10.1016/S0018-506X(02)00017-X 12614649 10.1016/s0018-506x(02)00017-x

[CR54] La Rosa, C., Cavallo, F., Pecora, A., Chincarini, M., Ala, U., Faulkes, C. G., Nacher, J., Cozzi, B., Sherwood, C. C., Amrein, I., & Bonfanti, L. (2020). Phylogenetic variation in cortical layer II immature neuron reservoir of mammals. *eLife,**9*, Article e55456. 10.7554/eLife.55456 32690132 10.7554/eLife.55456 PMC7373429

[CR55] Libby, P. (2015). Murine “model” Monotheism: An Iconoclast at the Altar of Mouse. *Circulation Research,**117*(11), 921–925. 10.1161/CIRCRESAHA.115.307523 26541681 10.1161/CIRCRESAHA.115.307523

[CR56] Lind, N. M., Moustgaard, A., Jelsing, J., Vajta, G., Cumming, P., & Hansen, A. K. (2007). The use of pigs in neuroscience: Modeling brain disorders. *Neuroscience and Biobehavioral Reviews,**31*(5), 728–751. 10.1016/j.neubiorev.2007.02.003 17445892 10.1016/j.neubiorev.2007.02.003

[CR57] Low, V. F., Faull, R. L. M., Bennet, L., Gunn, A. J., & Curtis, M. A. (2013). Neurogenesis and progenitor cell distribution in the subgranular zone and subventricular zone of the adult sheep brain. *Neuroscience,**244*, 173–187. 10.1016/j.neuroscience.2013.04.006 23587842 10.1016/j.neuroscience.2013.04.006

[CR58] Lucas, M. E., Hemsworth, L. M., & Hemsworth, P. H. (2024). Review: Early life piglet experiences and impacts on immediate and longer-term adaptability. *Animal: An International Journal of Animal Bioscience*, *18 Suppl 1*, 100889. 10.1016/j.animal.2023.100889 10.1016/j.animal.2023.100889 37468352

[CR59] Makowska, I. J., & Weary, D. M. (2016). Differences in anticipatory behaviour between rats (*Rattus norvegicus*) housed in standard versus semi-naturalistic laboratory environments. *PLoS One,**11*(1), Article e0147595. 10.1371/journal.pone.0147595 26820978 10.1371/journal.pone.0147595 PMC4731070

[CR60] Martin, B., Ji, S., Maudsley, S., & Mattson, M. P. (2010). “Control” laboratory rodents are metabolically morbid: Why it matters. *Proceedings of the National Academy of Sciences of the United States of America,**107*(14), 6127–6133. 10.1073/pnas.0912955107 20194732 10.1073/pnas.0912955107 PMC2852022

[CR61] Martinez-Ramirez, L., Slate, A., Price, G. D., Duhaime, A.-C., Staley, K. J., & Costine-Bartell, B. A. (2022). Robust, long-term video EEG monitoring in a porcine model of post-traumatic epilepsy. *eNeuro*. 10.1523/ENEURO.0025-22.2022 35697513 10.1523/ENEURO.0025-22.2022 PMC9275145

[CR62] McBride, J. H., & Monson, T. A. (2024). The evolution of primate litter size. *Humans,**4*(3), Article Article 3. 10.3390/humans4030014

[CR63] McBride, S. D., & Morton, A. J. (2018). Indices of comparative cognition: Assessing animal models of human brain function. *Experimental Brain Research,**236*(12), 3379–3390. 10.1007/s00221-018-5370-8 30267138 10.1007/s00221-018-5370-8 PMC6267686

[CR64] McBride, S. D., Perentos, N., & Morton, A. J. (2016). A mobile, high-throughput semi-automated system for testing cognition in large non-primate animal models of Huntington disease. *Journal of Neuroscience Methods,**265*, 25–33. 10.1016/j.jneumeth.2015.08.025 26327320 10.1016/j.jneumeth.2015.08.025

[CR65] Mehra, M., Henninger, N., Hirsch, J. A., Chueh, J., Wakhloo, A. K., & Gounis, M. J. (2012). Preclinical acute ischemic stroke modeling. *Journal of Neurointerventional Surgery,**4*(4), 307–313. 10.1136/neurintsurg-2011-010101 21990535 10.1136/neurintsurg-2011-010101

[CR66] Mendl, M., & Paul, E. S. (2020). Animal affect and decision-making. *Neuroscience and Biobehavioral Reviews,**112*, 144–163. 10.1016/j.neubiorev.2020.01.025 31991192 10.1016/j.neubiorev.2020.01.025

[CR67] Mental Illness—National Institute of Mental Health (NIMH). (2024). Retrieved August 27, 2024, from https://www.nimh.nih.gov/health/statistics/mental-illness

[CR68] Miller, C. T., Freiwald, W. A., Leopold, D. A., Mitchell, J. F., Silva, A. C., & Wang, X. (2016). Marmosets: A neuroscientific model of human social behavior. *Neuron,**90*(2), 219. 10.1016/j.neuron.2016.03.018 27100195 10.1016/j.neuron.2016.03.018 PMC4840471

[CR69] *Mini farm animals are adorable. There’s also a growing demand for them*. (2024, August 12). AP News. https://apnews.com/article/mini-farm-animals-cows-goat-9a62536eb3c679ff3bf0156cd20b9354

[CR70] Mitchell, S. J., Scheibye-Knudsen, M., Longo, D. L., & Cabo, Rde. (2015). Animal models of aging research: Implications for human aging and age-related diseases*. *Annual Review of Animal Biosciences,**3*(1), 283–303. 10.1146/annurev-animal-022114-110829 25689319 10.1146/annurev-animal-022114-110829

[CR71] Monteggia, L. M., Heimer, H., & Nestler, E. J. (2018). Meeting report: Can we make animal models of human mental illness? *Biological Psychiatry,**84*(7), 542–545. 10.1016/j.biopsych.2018.02.010 29606372 10.1016/j.biopsych.2018.02.010 PMC6269650

[CR72] Monticello, T. M., Jones, T. W., Dambach, D. M., Potter, D. M., Bolt, M. W., Liu, M., Keller, D. A., Hart, T. K., & Kadambi, V. J. (2017). Current nonclinical testing paradigm enables safe entry to first-in-human clinical trials: The IQ consortium nonclinical to clinical translational database. *Toxicology and Applied Pharmacology,**334*, 100–109. 10.1016/j.taap.2017.09.006 28893587 10.1016/j.taap.2017.09.006

[CR73] Morton, A. J. (2018). Large-Brained Animal Models of Huntington’s Disease: Sheep. *Methods in Molecular Biology (Clifton, N.J.)*, *1780*, 221–239. 10.1007/978-1-4939-7825-0_12 10.1007/978-1-4939-7825-0_12 29856022

[CR74] Morton, A. J., & Howland, D. S. (2013). Large genetic animal models of Huntington’s disease. *Journal of Huntington’s Disease,**2*(1), 3–19. 10.3233/JHD-130050 25063426 10.3233/JHD-130050

[CR75] Murphy, E., Nordquist, R. E., & van der Staay, F. J. (2014). A review of behavioural methods to study emotion and mood in pigs, *Sus scrofa*. *Applied Animal Behaviour Science,**159*, 9–28. 10.1016/j.applanim.2014.08.002

[CR76] Namdari, R., Jones, K., Chuang, S. S., Van Cruchten, S., Dincer, Z., Downes, N., Mikkelsen, L. F., Harding, J., Jäckel, S., Jacobsen, B., Kinyamu-Akunda, J., Lortie, A., Mhedhbi, S., Mohr, S., Schmitt, M. W., & Prior, H. (2021). Species selection for nonclinical safety assessment of drug candidates: Examples of current industry practice. *Regulatory Toxicology and Pharmacology,**126*, Article 105029. 10.1016/j.yrtph.2021.105029 34455009 10.1016/j.yrtph.2021.105029

[CR77] Nawroth, C., & Langbein, J. (Eds.). (2019). *Advances and Perspectives in Farm Animal Learning and Cognition*. Frontiers Media SA. 10.3389/978-2-88963-054-7 10.3389/fvets.2019.00172 PMC6558159 31214609

[CR78] Nawroth, C., Langbein, J., Coulon, M., Gabor, V., Oesterwind, S., Benz-Schwarzburg, J., & von Borell, E. (2019). Farm animal cognition—linking behavior, welfare and ethics. *Frontiers in Veterinary Science*. 10.3389/fvets.2019.00024 30838218 10.3389/fvets.2019.00024 PMC6383588

[CR79] Nestler, E. J., & Hyman, S. E. (2010). Animal models of neuropsychiatric disorders. *Nature Neuroscience,**13*(10), 1161–1169. 10.1038/nn.2647 20877280 10.1038/nn.2647 PMC3750731

[CR80] Netzley, A. H., & Pelled, G. (2023). The pig as a translational animal model for biobehavioral and neurotrauma research. *Biomedicines,**11*(8), 2165. 10.3390/biomedicines11082165 37626662 10.3390/biomedicines11082165 PMC10452425

[CR81] *Neuralink: Elon Musk unveils pig with chip in its brain*. (2020, August 29). https://www.bbc.com/news/world-us-canada-53956683

[CR82] Neziri, S., Köseoğlu, A. E., Deniz Köseoğlu, G., Özgültekin, B., & Özgentürk, N. Ö. (2024). Animal models in neuroscience with alternative approaches: Evolutionary, biomedical, and ethical perspectives. *Animal Models and Experimental Medicine,**7*(6), 868–880. 10.1002/ame2.12487 39375824 10.1002/ame2.12487 PMC11680486

[CR83] Nordquist, R. E. (2021). Behavioural tests for learning and cognition in humans and animals. In I. Camerlink (Ed.), *Bridging research disciplines to advance animal welfare science: A practical guide* (pp. 141–156). CABI. 10.1079/9781789247893.0009

[CR84] Nordquist, R. E., Meijer, E., van der Staay, F. J., & Arndt, S. S. (2017). Chapter 39—Pigs as Model Species to Investigate Effects of Early Life Events on Later Behavioral and Neurological Functions. In P. M. Conn (Ed.), *Animal Models for the Study of Human Disease (Second Edition)* (pp. 1003–1030). Academic Press. 10.1016/B978-0-12-809468-6.00039-5

[CR85] Ormandy, E. H., & Schuppli, C. A. (2014). Public attitudes toward animal research: A review. *Animals,**4*(3), 391–408. 10.3390/ani4030391 26480314 10.3390/ani4030391 PMC4494309

[CR86] Pardo, C. E., Kreuzer, M., & Bee, G. (2013). Effect of average litter weight in pigs on growth performance, carcass characteristics and meat quality of the offspring as depending on birth weight. *Animal,**7*(11), 1884–1892. 10.1017/S1751731113001419 23896082 10.1017/S1751731113001419

[CR87] Peacock, L., & Gerlach, J. (1999). New and old antipsychotics versus clozapine in a monkey model: Adverse effects and antiamphetamine effects. *Psychopharmacology,**144*(3), 189–197. 10.1007/s002130050993 10435384 10.1007/s002130050993

[CR88] Peacock, L., Hansen, L., Mørkeberg, F., & Gerlach, J. (1999). Chronic dopamine D1, dopamine D2 and combined dopamine D1 and D2 antagonist treatment in *Cebus apella* monkeys: Antiamphetamine effects and extrapyramidal side effects. *Neuropsychopharmacology: Official Publication of the American College of Neuropsychopharmacology,**20*(1), 35–43. 10.1016/S0893-133X(98)00049-9 9885783 10.1016/S0893-133X(98)00049-9

[CR89] Phillips, K. A., Bales, K. L., Capitanio, J. P., Conley, A., Czoty, P. W., ‘t Hart, B. A., Hopkins, W. D., Hu, S.-L., Miller, L. A., Nader, M. A., Nathanielsz, P. W., Rogers, J., Shively, C. A., & Voytko, M. L. (2014). Why primate models matter: Why Primate Models Matter. *American Journal of Primatology*, *76*(9), 801–827. 10.1002/ajp.22281 10.1002/ajp.22281 PMC4145602 24723482

[CR90] Piumatti, M., Palazzo, O., La Rosa, C., Crociara, P., Parolisi, R., Luzzati, F., Lévy, F., & Bonfanti, L. (2018). Non-Newly Generated, “Immature” Neurons in the Sheep Brain Are Not Restricted to Cerebral Cortex. *The Journal of Neuroscience,**38*(4), 826–842. 10.1523/JNEUROSCI.1781-17.2017 29217680 10.1523/JNEUROSCI.1781-17.2017 PMC6596233

[CR91] Pluchot, C., Adriaensen, H., Parias, C., Dubreuil, D., Arnould, C., Chaillou, E., & Love, S. A. (2024). Sheep (*Ovis aries*) training protocol for voluntary awake and unrestrained structural brain MRI acquisitions. *Behavior Research Methods*. 10.3758/s13428-024-02449-6 38907122 10.3758/s13428-024-02449-6 PMC11362526

[CR92] Polejaeva, I. A., Rutigliano, H. M., & Wells, K. D. (2016). Livestock in biomedical research: History, current status and future prospective. *Reproduction, Fertility and Development,**28*(2), 112. 10.1071/RD15343 27062879 10.1071/RD15343

[CR93] Prior, H., Haworth, R., Labram, B., Roberts, R., Wolfreys, A., & Sewell, F. (2021). Justification for species selection for pharmaceutical toxicity studies. *Toxicology Research,**9*(6), 758–770. 10.1093/toxres/tfaa081 10.1093/toxres/tfaa081 PMC7786171 33442468

[CR94] Puścian, A., & Knapska, E. (2022). Blueprints for measuring natural behavior. *IScience*. 10.1016/j.isci.2022.104635 35800771 10.1016/j.isci.2022.104635 PMC9254349

[CR95] Renggaman, A., Choi, H. L., Sudiarto, S. I., Alasaarela, L., & Nam, O. S. (2015). Development of pig welfare assessment protocol integrating animal-, environment-, and management-based measures. *Journal of Animal Science and Technology,**57*(1), 1. 10.1186/s40781-014-0034-0 26290721 10.1186/s40781-014-0034-0 PMC4540295

[CR96] Report from the Commission to the Council and the European Parliament Seventh Report on the Statistics on the Number of Animals Used for Experimental and Other Scientific Purposes in the Member States of the European Union (2013). https://eur-lex.europa.eu/legal-content/EN/TXT/?uri=CELEX%3A52013DC0859

[CR97] *Research Facility Annual Usage Summary Report | Animal and Plant Health Inspection Service*. (2023). https://www.aphis.usda.gov/awa/research-facility-report/annual-summary

[CR98] *Research Using Agricultural Animal Species | OLAW*. (n.d.). Retrieved May 2, 2025, from https://olaw.nih.gov/education/educational-resources/webinar-2024-03-21.htm

[CR99] Roberts, R. M., Smith, G. W., Bazer, F. W., Cibelli, J., Seidel, G. E., Bauman, D. E., Reynolds, L. P., & Ireland, J. J. (2009). Farm animal research in crisis. *Science,**324*(5926), 468–469. 10.1126/science.1168521 19390030 10.1126/science.1168521

[CR100] Roelofs, S., Boleij, H., Nordquist, R. E., & van der Staay, F. J. (2016). Making Decisions under Ambiguity: Judgment Bias Tasks for Assessing Emotional State in Animals. *Frontiers in Behavioral Neuroscience*, *10*. 10.3389/fnbeh.2016.00119 10.3389/fnbeh.2016.00119 PMC4899464 27375454

[CR101] Rush, A. J., Trivedi, M. H., Wisniewski, S. R., Nierenberg, A. A., Stewart, J. W., Warden, D., Niederehe, G., Thase, M. E., Lavori, P. W., Lebowitz, B. D., McGrath, P. J., Rosenbaum, J. F., Sackeim, H. A., Kupfer, D. J., Luther, J., & Fava, M. (2006). Acute and longer-term outcomes in depressed outpatients requiring one or several treatment steps: A STAR*D report. *The American Journal of Psychiatry,**163*(11), 1905–1917. 10.1176/ajp.2006.163.11.1905 17074942 10.1176/ajp.2006.163.11.1905

[CR102] Russell, W. M. S., & Burch, R. L. (1959/1992). *The principles of humane experimental technique*. Universities Federation for Animal Welfare. Retrieved November 13, 2024, from https://caat.jhsph.edu/the-principles-of-humane-experimental-technique/

[CR103] Schook, L., Beattie, C., Beever, J., Donovan, S., Jamison, R., Zuckermann, F., Niemi, S., Rothschild, M., Rutherford, M., & Smith, D. (2005). Swine in biomedical research: Creating the building blocks of animal models. *Animal Biotechnology,**16*(2), 183–190. 10.1080/10495390500265034 16342425 10.1080/10495390500265034

[CR104] Schroeder, D., Cook, J., Hirsch, F., Fenet, S., spsampsps Muthuswamy, V. (Eds.). (2018). *Ethics Dumping: Case Studies from North-South Research Collaborations*. Springer International Publishing. 10.1007/978-3-319-64731-9

[CR105] Scott, J. T., & Bourne, J. A. (2022). Modelling behaviors relevant to brain disorders in the nonhuman primate: Are we there yet? *Progress in Neurobiology,**208*, Article 102183. 10.1016/j.pneurobio.2021.102183 34728308 10.1016/j.pneurobio.2021.102183

[CR106] Sena, E. S., van der Worp, H. B., Bath, P. M. W., Howells, D. W., & Macleod, M. R. (2010). Publication bias in reports of animal stroke studies leads to major overstatement of efficacy. *PLoS Biology,**8*(3), Article e1000344. 10.1371/journal.pbio.1000344 20361022 10.1371/journal.pbio.1000344 PMC2846857

[CR107] Sewell, F., Edwards, J., Prior, H., & Robinson, S. (2016). Opportunities to apply the 3Rs in safety assessment programs. *ILAR Journal,**57*(2), 234–245. 10.1093/ilar/ilw024 28053076 10.1093/ilar/ilw024 PMC5886346

[CR108] Shemesh, Y., & Chen, A. (2023). A paradigm shift in translational psychiatry through rodent neuroethology. *Molecular Psychiatry,**28*(3), 993–1003. 10.1038/s41380-022-01913-z 36635579 10.1038/s41380-022-01913-z PMC10005947

[CR109] Shettleworth, S. J. (2010). *Cognition, evolution, and behavior, 2nd ed* (pp. xiii, 700). Oxford University Press.

[CR110] Simchick, G., Shen, A., Campbell, B., Park, H. J., West, F. D., & Zhao, Q. (2019). Pig brains have homologous resting-state networks with human brains. *Brain Connectivity,**9*(7), 566. 10.1089/brain.2019.0673 31115245 10.1089/brain.2019.0673 PMC6727477

[CR111] Simianer, H., & Köhn, F. (2010). Genetic management of the Göttingen Minipig population. *Journal of Pharmacological and Toxicological Methods,**62*(3), 221–226. 10.1016/j.vascn.2010.05.004 20570747 10.1016/j.vascn.2010.05.004

[CR112] Strawn, M., & Behura, S. K. (2022). Epigenetic regulation of fetal brain development in pig. *Gene,**844*, Article 146823. 10.1016/j.gene.2022.146823 35988784 10.1016/j.gene.2022.146823

[CR113] Sueur, C., Jacobs, A., Amblard, F., Petit, O., & King, A. J. (2011). How can social network analysis improve the study of primate behavior? *American Journal of Primatology,**73*(8), 703–719. 10.1002/ajp.20915 21181869 10.1002/ajp.20915

[CR114] Sutkus, L. T., Li, Z., & Dilger, R. N. (2025). Establishing the pig as a translational animal model for neurodevelopment. *Translational Neuroscience,**16*(1), 20250369. 10.1515/tnsci-2025-0369 40292422 10.1515/tnsci-2025-0369 PMC12032983

[CR115] Tanila, H. (2018). Testing cognitive functions in rodent disease models: Present pitfalls and future perspectives. *Behavioural Brain Research,**352*, 23–27. 10.1016/j.bbr.2017.05.040 28527690 10.1016/j.bbr.2017.05.040

[CR116] Tellam, R. L., Lemay, D. G., van Tassell, C. P., Lewin, H. A., Worley, K. C., & Elsik, C. G. (2009). Unlocking the bovine genome. *Bmc Genomics,**10*(1), Article 193. 10.1186/1471-2164-10-193 19393070 10.1186/1471-2164-10-193 PMC2680899

[CR117] Teshome, A. A., Abebe, E. C., Mengstie, M. A., Seid, M. A., Yitbarek, G. Y., Molla, Y. M., Baye, N. D., Yazie, T. S., Ayehu, G. W., & Taye, M. J. (2023). Post-traumatic stress disorder and associated factors among adult war survivors in Northwest Ethiopia: Community-based, cross-sectional study. *Frontiers in Psychiatry,**14*, 1083138. 10.3389/fpsyt.2023.1083138 37113553 10.3389/fpsyt.2023.1083138 PMC10126353

[CR118] Thapar, A., & Riglin, L. (2020). The importance of a developmental perspective in psychiatry: What do recent genetic-epidemiological findings show? *Molecular Psychiatry,**25*(8), 1631–1639. 10.1038/s41380-020-0648-1 31959848 10.1038/s41380-020-0648-1 PMC7387296

[CR119] USDA, National Agricultural Statistics Service (NASS). (2024a). *Cattle Inventory* (ISSN: 1948-9099). United States Department of Agriculture (USDA). https://www.nass.usda.gov/Publications/Todays_Reports/reports/cattle_inventory_2024.pdf

[CR120] USDA, National Agricultural Statistics Service (NASS). (2024b). *Livestock Slaughter 2023 Summary* [ISSN: 0499-0544]. United States Department of Agriculture (USDA). https://www.nass.usda.gov/Publications/Todays_Reports/reports/shep0120.pdf

[CR121] USDA, National Agricultural Statistics Service (NASS). (2024c). *Sheep and Goats Inventory 2023* (ISSN: 1949-1611). United States Department of Agriculture (USDA).

[CR122] van der Staay, F. J. (2006). Animal models of behavioral dysfunctions: Basic concepts and classifications, and an evaluation strategy. *Brain Research Reviews,**52*(1), 131–159. 10.1016/j.brainresrev.2006.01.006 16529820 10.1016/j.brainresrev.2006.01.006

[CR123] van der Staay, F. J., Arndt, S. S., & Nordquist, R. E. (2009). Evaluation of animal models of neurobehavioral disorders. *Behavioral and Brain Functions,**5*(1), 11. 10.1186/1744-9081-5-11 19243583 10.1186/1744-9081-5-11 PMC2669803

[CR124] van der Staay, F. J., Arndt, S. S., & Nordquist, R. E. (2010). The standardization-generalization dilemma: A way out. *Genes, Brain, and Behavior,**9*(8), 849–855. 10.1111/j.1601-183X.2010.00628.x 20662940 10.1111/j.1601-183X.2010.00628.x

[CR125] van der Staay, F. J., Nordquist, R. E., & Arndt, S. S. (2017). Chapter 3 - Large Farm Animal Models of Human Neurobehavioral and Psychiatric Disorders: Methodological and Practical Considerations. In P. M. Conn (Ed.), *Animal Models for the Study of Human Disease (Second Edition)* (pp. 71–100). Academic Press. 10.1016/B978-0-12-809468-6.00003-6

[CR126] van der Staay, F. J., Schuurman, T., Hulst, M., Smits, M., Prickaerts, J., Kenis, G., & Korte, S. M. (2010). Effects of chronic stress: A comparison between tethered and loose sows. *Physiology & Behavior,**100*(2), 154–164. 10.1016/j.physbeh.2010.02.020 20193701 10.1016/j.physbeh.2010.02.020

[CR127] Vink, R. (2018). Large animal models of traumatic brain injury. *Journal of Neuroscience Research,**96*(4), 527–535. 10.1002/jnr.24079 28500771 10.1002/jnr.24079

[CR128] Vrselja, Z., Daniele, S. G., Silbereis, J., Talpo, F., Morozov, Y. M., Sousa, A. M. M., Tanaka, B. S., Skarica, M., Pletikos, M., Kaur, N., Zhuang, Z. W., Liu, Z., Alkawadri, R., Sinusas, A. J., Latham, S. R., Waxman, S. G., & Sestan, N. (2019). Restoration of brain circulation and cellular functions hours post-mortem. *Nature,**568*(7752), 336–343. 10.1038/s41586-019-1099-1 30996318 10.1038/s41586-019-1099-1 PMC6844189

[CR129] Walters, E. M., Wells, K. D., Bryda, E. C., Schommer, S., & Prather, R. S. (2017). Swine models, genomic tools and services to enhance our understanding of human health and diseases. *Lab Animal,**46*(4), 167–172. 10.1038/laban.1215 28328880 10.1038/laban.1215 PMC7091812

[CR130] Webster, J. (2011). Zoomorphism and anthropomorphism: Fruitful fallacies? *Animal Welfare,**20*(1), 29–36. 10.1017/S0962728600002402

[CR131] Weerts, E. M., Fantegrossi, W. E., & Goodwin, A. K. (2007). The value of nonhuman primates in drug abuse research. *Experimental and Clinical Psychopharmacology,**15*(4), 309–327. 10.1037/1064-1297.15.4.309 17696678 10.1037/1064-1297.15.4.309

[CR132] Wernersson, R., Schierup, M. H., Jørgensen, F. G., Gorodkin, J., Panitz, F., Stærfeldt, H.-H., Christensen, O. F., Mailund, T., Hornshøj, H., Klein, A., Wang, J., Liu, B., Hu, S., Dong, W., Li, W., Wong, G. K., Yu, J., Wang, J., Bendixen, C., … Bolund, L. (2005). Pigs in sequence space: A 0.66X coverage pig genome survey based on shotgun sequencing. *BMC Genomics*, *6*(1), 70. 10.1186/1471-2164-6-70 10.1186/1471-2164-6-70 PMC1142312 15885146

[CR133] Xiong, Y., Mahmood, A., & Chopp, M. (2013). Animal models of traumatic brain injury. *Nature Reviews Neuroscience,**14*(2), 128–142. 10.1038/nrn3407 23329160 10.1038/nrn3407 PMC3951995

[CR134] Yao, Q., Cheng, S., Pan, Q., Yu, J., Cao, G., Li, L., & Cao, H. (2024). Organoids: Development and applications in disease models, drug discovery, precision medicine, and regenerative medicine. *MedComm (London),**5*(10), Article e735. 10.1002/mco2.735 10.1002/mco2.735 PMC11416091 39309690

[CR135] Żakowski, W. (2020). Animal use in neurobiological research. *Neuroscience,**433*, 1–10. 10.1016/j.neuroscience.2020.02.049 32156550 10.1016/j.neuroscience.2020.02.049

