# Supplementary material for: Incorporating farm animal models for the study of neuropsychiatric diseases: Expansion of the possibilities
Source: Cogn Affect Behav Neurosci. 2025 Aug 7;26(1):16–32. doi: 10.3758/s13415-025-01332-x (PMC12847119; doi:10.3758/s13415-025-01332-x)
Supplement: Supplementary file 1 — Supplementary file1 (DOCX 124 KB) [file 13415_2025_1332_MOESM1_ESM.docx]

**Supplementary material**

**Incorporating farm animal models for the study of neuropsychiatric diseases**

Alexandra K. Dwulit^1,2,3^, Rajendra A. Morey^3,6,7^, F. Josef van der Staay^4,5^

^1^Department of Molecular Biomedical Sciences, North Carolina State University College of Veterinary Medicine, Raleigh, NC, USA

^2^Comparative Medicine Institute, North Carolina State University College of Veterinary Medicine, Raleigh, NC, USA

^3^Duke-UNC Brain Imaging and Analysis Center, Duke University, Durham, NC, USA

^4^Department of Population Health Sciences, Division of Farm Animal Health, Behaviour and Welfare Group, Faculty of Veterinary Medicine, University Utrecht, Utrecht, The Netherlands

^5^University Medical Center Utrecht (UMCU) Brain Center, Utrecht, The Netherlands

^6^Department of Psychiatry and Behavioral Sciences, Duke University, Durham, NC, USA

^7^Mental Illness Research Education and Clinical Center for Post Deployment Mental Health, Durham VA Medical Center, Durham, NC, USA

Further materials are provided for tests to measure cognition (physical, social) and affect, as well as validated tests (brain atlases, genetic manipulation) in farm animal models. Examples of studies that used farm animal neuropsychiatric disease models are also provided. This is not meant as a comprehensive summary of all available literature.

**Table S1:** Validated tests in pigs, sheep, goats, and cows.

**Table S2:** Tests to measure cognition and affect in pigs, sheep, goats, cows, and horses.

**Table S1:** *Validated tests in pigs, sheep, goats, and cows*. This is not a review of all validated methods, but provides an overview of what is currently available.

| **Validated tests** | **Species** |  |  |  |
| --- | --- | --- | --- | --- |
|  | **Pigs** | **Sheep** | **Goats** | **Cows** |
| **Brain atlases (high resolution, 3D stereotaxic)** | Göttingen minipig, (Andersen et al., 2005); neonatal pig (Conrad et al., 2014); commercial pig (Saikali et al., 2010) | Nitzsche et al., 2015 | Sunagawa et al., 2015 | Saito et al., 2004 |
| **Behavior and cognition** | Forkman et al., 2007; Gieling et al., 2011; Netzley & Pelled, 2023; Nordquist et al., 2017 | Doyle, 2017; Forkman et al., 2007 | Nawroth et al., 2019 | Forkman et al., 2007; Lagisz et al., 2020 |
| **Genetic manipulation** | Aigner et al., 2010; Bähr & Wolf, 2012; Klymiuk et al., 2012 | Kalds et al., 2019 | Kalds et al., 2019 | Yum et al., 2018 |
| **Neuropsychiatric disease models** | Huntington's disease, (Baxa et al., 2013); Parkinson's and Alzheimer's disease, (Holm et al., 2016); TBI (Netzley & Pelled, 2023); Chronic stress and depression (Cocchi et al., 2009; Lind et al., 2007; van der Staay et al., 2010); Autism spectrum disorder (Qiu et al., 2024); Neurodevelopmental disorders (Netzley & Pelled, 2023); Anxiety, schizophrenia, aggression (Lind et al., 2007) | Huntington's disease, (Jacobsen et al., 2010); CLN1 Batten disease (Murray & Mitchell, 2022); TBI and chronic traumatic encephalopathy (Ackermans et al., 2021) | TBI (Ackermans et al., 2021) | Alzheimer's disease, (Moreno-Gonzalez et al., 2021); Bovine spongiform encephalopathy (Asher & Gregori, 2018) |

**Table S2**: *Tests to measure cognition and affect in pigs, sheep, goats, cows, and horses.* This is not a review of the available literature. It lists only examples of studies that have been conducted in the various areas.

| **Testing domain** | **Subdomains** | **Species** |  |  |  |  |
| --- | --- | --- | --- | --- | --- | --- |
|  |  | **Pigs** | **Sheep** | **Goats** | **Cows** | **Horses** |
| **Physical cognition** | Categorization | Wondrak et al., 2018 | Ginane & Dumont, 2010 | Meyer et al., 2012 | Coulon et al., 2011 | Hanggi, 1999; Hanggi & Ingersoll, 2009 |
|  | Numerical ability | NA | Quail & Fraser, 2024 | Quail & Fraser, 2024 | NA | Gabor & Gerken, 2014; Uller & Lewis, 2009 |
|  | Object permanence | Nawroth, Ebersbach, et al., 2014; Nawroth et al., 2013 | Quail & Fraser, 2024 | Nawroth et al., 2015b | NA | Krueger et al., 2011; Proops & McComb, 2010 |
|  | Reasoning/inferences | Nawroth & von Borell, 2015 | Nawroth, Borell, et al., 2014 | Nawroth, Borell, et al., 2014 | NA | NA |
|  | Problem-solving abilities | Netzley & Pelled, 2023 | Coulon et al., 2015; Doyle et al., 2014 | Raoult et al., 2021; Rosenberger et al., 2021 | Stenfelt et al., 2022 | Esch et al., 2019 |
|  | Spatial memory and maze tests | Grimberg-Henrici et al., 2016; van der Staay et al., 2012 | Doyle, 2017; Doyle et al., 2014 | Raoult et al., 2021 | Stenfelt et al., 2022 | McLean, 2004 |
| **Social cognition** | Individual recognition and discrimination (conspecifics or humans) | McLeman et al., 2005, 2008 | Kendrick et al., 2001; Knolle et al., 2017 | Keil et al., 2012 | Coulon et al., 2007, 2011; Rybarczyk et al., 2001 | Krueger et al., 2011; Proops et al., 2009 |
|  | Social learning (vertical, horizontal, from humans) | Figueroa et al., 2013; Oostindjer et al., 2011; Veit et al., 2017 | NA | Baciadonna et al., 2013; Briefer et al., 2014; Glasser et al., 2009; Nawroth et al., 2016 | Bailey et al., 2000; Howery et al., 1998; Veissier, 1993 | Burla et al., 2018; Krueger et al., 2014; Rørvang et al., 2015; Schuetz et al., 2017 |
|  | Attribution of attention | Held et al., 2001; Nawroth et al., 2013 | Monk et al., 2023 | Nawroth et al., 2015a; Nawroth & McElligott, 2017 | Kremer et al., 2021 | Proops & McComb, 2010 |
| **Affect** | Cognitive bias tests | Ede & Parsons, 2023 | Doyle et al., 2010; Lee et al., 2016 | Baciadonna et al., 2016) | Kremer et al., 2021 | Henry et al., 2017 |
|  | Vocalization analysis | Briefer et al., 2022 | Papadaki et al., 2021 | Briefer et al., 2015 | Laurijs et al., 2021 | Briefer et al., 2017 |
|  | Fear and anxiety tests | Dalmau et al., 2009; Haigh et al., 2020 | Lee et al., 2016 | Rosenberger et al., 2022 | de Passillé et al., 1995; Kilgour et al., 2006 | Visser et al., 2002 |

**Supplementary References**

Ackermans, N. L., Varghese, M., Wicinski, B., Torres, J., De Gasperi, R., Pryor, D., Elder, G. A., Gama Sosa, M. A., Reidenberg, J. S., Williams, T. M., & Hof, P. R. (2021). Unconventional animal models for traumatic brain injury and chronic traumatic encephalopathy. *Journal of Neuroscience Research*, *99*(10), 2463–2477. https://doi.org/10.1002/jnr.24920

Aigner, B., Renner, S., Kessler, B., Klymiuk, N., Kurome, M., Wünsch, A., & Wolf, E. (2010). Transgenic pigs as models for translational biomedical research. *Journal of Molecular Medicine*, *88*(7), 653–664. https://doi.org/10.1007/s00109-010-0610-9

Andersen, F., Watanabe, H., Bjarkam, C., Danielsen, E. H., & Cumming, P. (2005). Pig brain stereotaxic standard space: Mapping of cerebral blood flow normative values and effect of MPTP-lesioning. *Brain Research Bulletin*, *66*(1), 17–29. https://doi.org/10.1016/j.brainresbull.2005.02.033

Asher, D. M., & Gregori, L. (2018). Human transmissible spongiform encephalopathies: Historic view. *Handbook of Clinical Neurology*, *153*, 1–17. https://doi.org/10.1016/B978-0-444-63945-5.00001-5

Baciadonna, L., McElligott, A. G., & Briefer, E. F. (2013). Goats favour personal over social information in an experimental foraging task. *PeerJ*, *1*, e172. https://doi.org/10.7717/peerj.172

Baciadonna, L., Nawroth, C., & McElligott, A. G. (2016). Judgement bias in goats (Capra hircus): Investigating the effects of human grooming. *PeerJ*, *4*, e2485. https://doi.org/10.7717/peerj.2485

Bähr, A., & Wolf, E. (2012). Domestic Animal Models for Biomedical Research. *Reproduction in Domestic Animals*, *47*(s4), 59–71. https://doi.org/10.1111/j.1439-0531.2012.02056.x

Bailey, D. W., Howery, L. D., & Boss, D. L. (2000). Effects of social facilitation for locating feeding sites by cattle in an eight-arm radial maze. *Applied Animal Behaviour Science*, *68*(2), 93–105. https://doi.org/10.1016/S0168-1591(00)00091-5

Baxa, M., Hruska-Plochan, M., Juhas, S., Vodicka, P., Pavlok, A., Juhasova, J., Miyanohara, A., Nejime, T., Klima, J., Macakova, M., Marsala, S., Weiss, A., Kubickova, S., Musilova, P., Vrtel, R., Sontag, E. M., Thompson, L. M., Schier, J., Hansikova, H., … Motlik, J. (2013). A Transgenic Minipig Model of Huntington’s Disease. *Journal of Huntington’s Disease*, *2*(1), 47–68. https://doi.org/10.3233/JHD-130001

Briefer, E. F., Haque, S., Baciadonna, L., & McElligott, A. G. (2014). Goats excel at learning and remembering a highly novel cognitive task. *Frontiers in Zoology*, *11*(1), 20. https://doi.org/10.1186/1742-9994-11-20

Briefer, E. F., Mandel, R., Maigrot, A.-L., Briefer Freymond, S., Bachmann, I., & Hillmann, E. (2017). Perception of emotional valence in horse whinnies. *Frontiers in Zoology*, *14*(1), 8. https://doi.org/10.1186/s12983-017-0193-1

Briefer, E. F., Sypherd, C. C.-R., Linhart, P., Leliveld, L. M. C., Padilla de la Torre, M., Read, E. R., Guérin, C., Deiss, V., Monestier, C., Rasmussen, J. H., Špinka, M., Düpjan, S., Boissy, A., Janczak, A. M., Hillmann, E., & Tallet, C. (2022). Classification of pig calls produced from birth to slaughter according to their emotional valence and context of production. *Scientific Reports*, *12*(1), 3409. https://doi.org/10.1038/s41598-022-07174-8

Briefer, E. F., Tettamanti, F., & McElligott, A. G. (2015). Emotions in goats: Mapping physiological, behavioural and vocal profiles. *Animal Behaviour*, *99*, 131–143. https://doi.org/10.1016/j.anbehav.2014.11.002

Burla, J.-B., Siegwart, J., & Nawroth, C. (2018). Human Demonstration Does Not Facilitate the Performance of Horses (Equus caballus) in a Spatial Problem-Solving Task. *Animals*, *8*(6), Article 6. https://doi.org/10.3390/ani8060096

Cocchi, M., Sardi, L., Tonello, L., & Martelli, G. (2009). Do mood disorders play a role in pig welfare? *Italian Journal of Animal Science*, *8*(4), 691–704. https://doi.org/10.4081/ijas.2009.691

Conrad, M. S., Sutton, B. P., Dilger, R. N., & Johnson, R. W. (2014). An In Vivo Three-Dimensional Magnetic Resonance Imaging-Based Averaged Brain Collection of the Neonatal Piglet (Sus scrofa). *PLOS ONE*, *9*(9), e107650. https://doi.org/10.1371/journal.pone.0107650

Coulon, M., Baudoin, C., Heyman, Y., & Deputte, B. L. (2011). Cattle discriminate between familiar and unfamiliar conspecifics by using only head visual cues. *Animal Cognition*, *14*(2), 279–290. https://doi.org/10.1007/s10071-010-0361-6

Coulon, M., Deputte, B. L., Heyman, Y., Delatouche, L., Richard, C., & Baudoin, C. (2007). Visual discrimination by heifers (Bos taurus) of their own species. *Journal of Comparative Psychology*, *121*(2), 198–204. https://doi.org/10.1037/0735-7036.121.2.198

Coulon, M., Nowak, R., Andanson, S., Petit, B., Lévy, F., & Boissy, A. (2015). Effects of prenatal stress and emotional reactivity of the mother on emotional and cognitive abilities in lambs: Prenatal Stress in Sheep. *Developmental Psychobiology*, *57*(5), 626–636. https://doi.org/10.1002/dev.21320

Dalmau, A., Fabrega, E., & Velarde, A. (2009). Fear assessment in pigs exposed to a novel object test. *Applied Animal Behaviour Science*, *117*(3), 173–180. https://doi.org/10.1016/j.applanim.2008.12.014

de Passillé, A. M., Rushen, J., & Martin, F. (1995). Interpreting behaviour of calves in an open-field test: A factor analysis. *Applied Animal Behaviour Science*, *45*(3–4), 201–213. https://doi.org/10.1016/0168-1591(95)00622-Y

Doyle, R. E. (2017). 4—Sheep cognition and its implications for welfare. In D. M. Ferguson, C. Lee, & A. Fisher (Eds.), *Advances in Sheep Welfare* (pp. 55–71). Woodhead Publishing. https://doi.org/10.1016/B978-0-08-100718-1.00004-2

Doyle, R. E., Freire, R., Cowling, A., Knott, S. A., & Lee, C. (2014). Performance of sheep in a spatial maze is impeded by negative stimuli. *Applied Animal Behaviour Science*, *151*, 36–42. https://doi.org/10.1016/j.applanim.2013.11.009

Doyle, R. E., Vidal, S., Hinch, G. N., Fisher, A. D., Boissy, A., & Lee, C. (2010). The effect of repeated testing on judgement biases in sheep. *Behavioural Processes*, *83*(3), 349–352. https://doi.org/10.1016/j.beproc.2010.01.019

Ede, T., & Parsons, T. D. (2023). Cognitive tasks as measures of pig welfare: A systematic review. *Frontiers in Veterinary Science*, *10*. https://doi.org/10.3389/fvets.2023.1251070

Esch, L., Wöhr, C., Erhard, M., & Krüger, K. (2019). Horses’ (Equus Caballus) Laterality, Stress Hormones, and Task Related Behavior in Innovative Problem-Solving. *Animals*, *9*(5), Article 5. https://doi.org/10.3390/ani9050265

Figueroa, J., Solà-Oriol, D., Manteca, X., & Pérez, J. F. (2013). Social learning of feeding behaviour in pigs: Effects of neophobia and familiarity with the demonstrator conspecific. *Applied Animal Behaviour Science*, *148*(1), 120–127. https://doi.org/10.1016/j.applanim.2013.06.002

Forkman, B., Boissy, A., Meunier-Salaün, M.-C., Canali, E., & Jones, R. B. (2007). A critical review of fear tests used on cattle, pigs, sheep, poultry and horses. *Physiology & Behavior*, *92*(3), 340–374. https://doi.org/10.1016/j.physbeh.2007.03.016

Gabor, V., & Gerken, M. (2014). Shetland ponies (Equus caballus) show quantity discrimination in a matching-to-sample design. *Animal Cognition*, *17*(6), 1233–1243. https://doi.org/10.1007/s10071-014-0753-0

Gieling, E. T., Schuurman, T., Nordquist, R. E., & van der Staay, F. J. (2011). The pig as a model animal for studying cognition and neurobehavioral disorders. *Current Topics in Behavioral Neurosciences*, *7*, 359–383. https://doi.org/10.1007/7854_2010_112

Ginane, C., & Dumont, B. (2010). Do grazing sheep use species-based categorization to select their diet? *Behavioural Processes*, *84*(2), 622–624. https://doi.org/10.1016/j.beproc.2010.01.022

Glasser, T. A., Ungar, E. D., Landau, S. Y., Perevolotsky, A., Muklada, H., & Walker, J. W. (2009). Breed and maternal effects on the intake of tannin-rich browse by juvenile domestic goats (Capra hircus). *Applied Animal Behaviour Science*, *119*(1–2), 71–77. https://doi.org/10.1016/j.applanim.2009.02.028

Grimberg-Henrici, C. G. E., Vermaak, P., Elizabeth Bolhuis, J., Nordquist, R. E., & van der Staay, F. J. (2016). Effects of environmental enrichment on cognitive performance of pigs in a spatial holeboard discrimination task. *Animal Cognition*, *19*(2), 271–283. https://doi.org/10.1007/s10071-015-0932-7

Haigh, A., Chou, J.-Y., & O’Driscoll, K. (2020). Variations in the Behavior of Pigs During an Open Field and Novel Object Test. *Frontiers in Veterinary Science*, *7*. https://doi.org/10.3389/fvets.2020.00607

Hanggi, E. B. (1999). Categorization learning in horses (Equus caballus). *Journal of Comparative Psychology*, *113*(3), 243–252. https://doi.org/10.1037/0735-7036.113.3.243

Hanggi, E. B., & Ingersoll, J. F. (2009). Long-term memory for categories and concepts in horses (Equus caballus). *Animal Cognition*, *12*(3), 451–462. https://doi.org/10.1007/s10071-008-0205-9

Held, S., Mendl, M., Devereux, C., & Byrne, R. W. (2001). Behaviour of Domestic Pigs in a Visual Perspective Taking Task. *Behaviour*, *138*(11/12), 1337–1354.

Henry, S., Fureix, C., Rowberry, R., Bateson, M., & Hausberger, M. (2017). Do horses with poor welfare show ‘pessimistic’ cognitive biases? *The Science of Nature*, *104*(1–2), 8. https://doi.org/10.1007/s00114-016-1429-1

Holm, I. E., Alstrup, A. K. O., & Luo, Y. (2016). Genetically modified pig models for neurodegenerative disorders. *The Journal of Pathology*, *238*(2), 267–287. https://doi.org/10.1002/path.4654

Howery, L. D., Provenza, F. D., Banner, R. E., & Scott, C. B. (1998). Social and environmental factors influence cattle distribution on rangeland. *Applied Animal Behaviour Science*, *55*(3–4), 231–244. https://doi.org/10.1016/S0168-1591(97)00054-3

Jacobsen, J. C., Bawden, C. S., Rudiger, S. R., McLaughlan, C. J., Reid, S. J., Waldvogel, H. J., MacDonald, M. E., Gusella, J. F., Walker, S. K., Kelly, J. M., Webb, G. C., Faull, R. L. M., Rees, M. I., & Snell, R. G. (2010). An ovine transgenic Huntington’s disease model. *Human Molecular Genetics*, *19*(10), 1873–1882. https://doi.org/10.1093/hmg/ddq063

Kalds, P., Zhou, S., Cai, B., Liu, J., Wang, Y., Petersen, B., Sonstegard, T., Wang, X., & Chen, Y. (2019). Sheep and Goat Genome Engineering: From Random Transgenesis to the CRISPR Era. *Frontiers in Genetics*, *10*, 750. https://doi.org/10.3389/fgene.2019.00750

Keil, N. M., Imfeld-Mueller, S., Aschwanden, J., & Wechsler, B. (2012). Are head cues necessary for goats (Capra hircus) in recognising group members? *Animal Cognition*, *15*(5), 913–921. https://doi.org/10.1007/s10071-012-0518-6

Kendrick, K. M., da Costa, A. P., Leigh, A. E., Hinton, M. R., & Peirce, J. W. (2001). Sheep don’t forget a face. *Nature*, *414*(6860), 165–166. https://doi.org/10.1038/35102669

Kilgour, R. J., Melville, G. J., & Greenwood, P. L. (2006). Individual differences in the reaction of beef cattle to situations involving social isolation, close proximity of humans, restraint and novelty. *Applied Animal Behaviour Science*, *99*(1), 21–40. https://doi.org/10.1016/j.applanim.2005.09.012

Klymiuk, N., Böcker, W., Schönitzer, V., Bähr, A., Radic, T., Fröhlich, T., Wünsch, A., Keßler, B., Kurome, M., Schilling, E., Herbach, N., Wanke, R., Nagashima, H., Mutschler, W., Arnold, G. J., Schwinzer, R., Schieker, M., & Wolf, E. (2012). First inducible transgene expression in porcine large animal models. *FASEB Journal: Official Publication of the Federation of American Societies for Experimental Biology*, *26*(3), 1086–1099. https://doi.org/10.1096/fj.11-185041

Knolle, F., McBride, S. D., Stewart, J. E., Goncalves, R. P., & Morton, A. J. (2017). A stop-signal task for sheep: Introduction and validation of a direct measure for the stop-signal reaction time. *Animal Cognition*, *20*(4), 615–626. https://doi.org/10.1007/s10071-017-1085-7

Kremer, L., Bus, J. D., Webb, L. E., Bokkers, E. A. M., Engel, B., van der Werf, J. T. N., Schnabel, S. K., & van Reenen, C. G. (2021). Housing and personality effects on judgement and attention biases in dairy cows. *Scientific Reports*, *11*(1), 22984. https://doi.org/10.1038/s41598-021-01843-w

Krueger, K., Farmer, K., & Heinze, J. (2014). The effects of age, rank and neophobia on social learning in horses. *Animal Cognition*, *17*(3), 645–655. https://doi.org/10.1007/s10071-013-0696-x

Krueger, K., Flauger, B., Farmer, K., & Maros, K. (2011). Horses (Equus caballus) use human local enhancement cues and adjust to human attention. *Animal Cognition*, *14*(2), 187–201. https://doi.org/10.1007/s10071-010-0352-7

Lagisz, M., Zidar, J., Nakagawa, S., Neville, V., Sorato, E., Paul, E. S., Bateson, M., Mendl, M., & Løvlie, H. (2020). Optimism, pessimism and judgement bias in animals: A systematic review and meta-analysis. *Neuroscience & Biobehavioral Reviews*, *118*, 3–17. https://doi.org/10.1016/j.neubiorev.2020.07.012

Laurijs, K. A., Briefer, E. F., Reimert, I., & Webb, L. E. (2021). Vocalisations in farm animals: A step towards positive welfare assessment. *Applied Animal Behaviour Science*, *236*, 105264. https://doi.org/10.1016/j.applanim.2021.105264

Lee, C., Verbeek, E., Doyle, R., & Bateson, M. (2016). Attention bias to threat indicates anxiety differences in sheep. *Biology Letters*, *12*(6), 20150977. https://doi.org/10.1098/rsbl.2015.0977

Lind, N. M., Moustgaard, A., Jelsing, J., Vajta, G., Cumming, P., & Hansen, A. K. (2007). The use of pigs in neuroscience: Modeling brain disorders. *Neuroscience & Biobehavioral Reviews*, *31*(5), 728–751. https://doi.org/10.1016/j.neubiorev.2007.02.003

McLean, A. N. (2004). Short-term spatial memory in the domestic horse. *Applied Animal Behaviour Science*, *85*(1), 93–105. https://doi.org/10.1016/j.applanim.2003.09.009

McLeman, M. A., Mendl, M., Jones, R. B., White, R., & Wathes, C. M. (2005). Discrimination of conspecifics by juvenile domestic pigs, Sus scrofa. *Animal Behaviour*, *70*(2), 451–461. https://doi.org/10.1016/j.anbehav.2004.11.013

McLeman, M. A., Mendl, M. T., Jones, R. B., & Wathes, C. M. (2008). Social discrimination of familiar conspecifics by juvenile pigs, Sus scrofa: Development of a non-invasive method to study the transmission of unimodal and bimodal cues between live stimuli. *Applied Animal Behaviour Science*, *115*(3–4), 123–137. https://doi.org/10.1016/j.applanim.2008.06.010

Meyer, S., Nürnberg, G., Puppe, B., & Langbein, J. (2012). The cognitive capabilities of farm animals: Categorisation learning in dwarf goats (Capra hircus). *Animal Cognition*, *15*(4), 567–576. https://doi.org/10.1007/s10071-012-0485-y

Monk, J. E., Colditz, I. G., Clark, S., & Lee, C. (2023). Repeatability of an attention bias test for sheep suggests variable influence of state and trait affect on behaviour. *PeerJ*, *11*, e14730. https://doi.org/10.7717/peerj.14730

Moreno-Gonzalez, I., Edwards, G., Morales, R., Duran-Aniotz, C., Escobedo, G., Marquez, M., Pumarola, M., & Soto, C. (2021). Aged Cattle Brain Displays Alzheimer’s Disease-Like Pathology and Promotes Brain Amyloidosis in a Transgenic Animal Model. *Frontiers in Aging Neuroscience*, *13*, 815361. https://doi.org/10.3389/fnagi.2021.815361

Murray, S. J., & Mitchell, N. L. (2022). The Translational Benefits of Sheep as Large Animal Models of Human Neurological Disorders. *Frontiers in Veterinary Science*, *9*, 831838. https://doi.org/10.3389/fvets.2022.831838

Nawroth, C., Baciadonna, L., & McElligott, A. G. (2016). Goats learn socially from humans in a spatial problem-solving task. *Animal Behaviour*, *121*, 123–129. https://doi.org/10.1016/j.anbehav.2016.09.004

Nawroth, C., Borell, E. von, & Langbein, J. (2014). Exclusion Performance in Dwarf Goats (Capra aegagrus hircus) and Sheep (Ovis orientalis aries). *PLOS ONE*, *9*(4), e93534. https://doi.org/10.1371/journal.pone.0093534

Nawroth, C., Ebersbach, M., & von Borell, E. (2013). A note on pigs’ knowledge of hidden objects. *Archives Animal Breeding*, *56*(1), 861–872. https://doi.org/10.7482/0003-9438-56-086

Nawroth, C., Ebersbach, M., & von Borell, E. (2014). Juvenile domestic pigs (Sus scrofa domestica) use human-given cues in an object choice task. *Animal Cognition*, *17*(3), 701–713. https://doi.org/10.1007/s10071-013-0702-3

Nawroth, C., Langbein, J., Coulon, M., Gabor, V., Oesterwind, S., Benz-Schwarzburg, J., & von Borell, E. (2019). Farm Animal Cognition—Linking Behavior, Welfare and Ethics. *Frontiers in Veterinary Science*, *6*. https://doi.org/10.3389/fvets.2019.00024

Nawroth, C., & McElligott, A. G. (2017). Human head orientation and eye visibility as indicators of attention for goats (Capra hircus). *PeerJ*, *5*, e3073. https://doi.org/10.7717/peerj.3073

Nawroth, C., & von Borell, E. (2015). Domestic pigs’ (Sus scrofa domestica) use of direct and indirect visual and auditory cues in an object choice task. *Animal Cognition*, *18*(3), 757–766. https://doi.org/10.1007/s10071-015-0842-8

Nawroth, C., von Borell, E., & Langbein, J. (2015a). ‘Goats that stare at men’: Dwarf goats alter their behaviour in response to human head orientation, but do not spontaneously use head direction as a cue in a food-related context. *Animal Cognition*, *18*(1), 65–73. https://doi.org/10.1007/s10071-014-0777-5

Nawroth, C., von Borell, E., & Langbein, J. (2015b). Object permanence in the dwarf goat (Capra aegagrus hircus): Perseveration errors and the tracking of complex movements of hidden objects. *Applied Animal Behaviour Science*, *167*, 20–26. https://doi.org/10.1016/j.applanim.2015.03.010

Netzley, A. H., & Pelled, G. (2023). The Pig as a Translational Animal Model for Biobehavioral and Neurotrauma Research. *Biomedicines*, *11*(8), 2165. https://doi.org/10.3390/biomedicines11082165

Nitzsche, B., Frey, S., Collins, L. D., Seeger, J., Lobsien, D., Dreyer, A., Kirsten, H., Stoffel, M. H., Fonov, V. S., & Boltze, J. (2015). A stereotaxic, population-averaged T1w ovine brain atlas including cerebral morphology and tissue volumes. *Frontiers in Neuroanatomy*, *9*. https://doi.org/10.3389/fnana.2015.00069

Nordquist, R. E., Meijer, E., van der Staay, F. J., & Arndt, S. S. (2017). Chapter 39—Pigs as Model Species to Investigate Effects of Early Life Events on Later Behavioral and Neurological Functions. In P. M. Conn (Ed.), *Animal Models for the Study of Human Disease (Second Edition)* (pp. 1003–1030). Academic Press. https://doi.org/10.1016/B978-0-12-809468-6.00039-5

Oostindjer, M., Bolhuis, J. E., Simon, K., Brand, H. van den, & Kemp, B. (2011). Perinatal Flavour Learning and Adaptation to Being Weaned: All the Pig Needs Is Smell. *PLOS ONE*, *6*(10), e25318. https://doi.org/10.1371/journal.pone.0025318

Papadaki, K., Laliotis, G. P., & Bizelis, I. (2021). Acoustic variables of high-pitched vocalizations in dairy sheep breeds. *Applied Animal Behaviour Science*, *241*, 105398. https://doi.org/10.1016/j.applanim.2021.105398

Proops, L., & McComb, K. (2010). Attributing attention: The use of human-given cues by domestic horses (Equus caballus). *Animal Cognition*, *13*(2), 197–205. https://doi.org/10.1007/s10071-009-0257-5

Proops, L., McComb, K., & Reby, D. (2009). Cross-modal individual recognition in domestic horses (Equus caballus). *Proceedings of the National Academy of Sciences*, *106*(3), 947–951. https://doi.org/10.1073/pnas.0809127105

Qiu, S., Jia, J., Xu, B., Wu, N., Cao, H., Xie, S., Cui, J., Ma, J., Pan, Y.-H., & Yuan, X.-B. (2024). Development and evaluation of an autism pig model. *Lab Animal*, *53*(12), 376–386. https://doi.org/10.1038/s41684-024-01475-3

Quail, M. R., & Fraser, M. D. (2024). Pulling the wool over their eyes? Object permanence, numerical competence and categorisation in alternative livestock species. *Applied Animal Behaviour Science*, *270*, 106131. https://doi.org/10.1016/j.applanim.2023.106131

Raoult, C. M. C., Osthaus, B., Hildebrand, A. C. G., McElligott, A. G., & Nawroth, C. (2021). Goats show higher behavioural flexibility than sheep in a spatial detour task. *Royal Society Open Science*, *8*(3), 201627. https://doi.org/10.1098/rsos.201627

Rørvang, M. V., Ahrendt, L. P., & Christensen, J. W. (2015). Horses fail to use social learning when solving spatial detour tasks. *Animal Cognition*, *18*(4), 847–854. https://doi.org/10.1007/s10071-015-0852-6

Rosenberger, K., Simmler, M., Langbein, J., Keil, N., & Nawroth, C. (2021). Performance of goats in a detour and a problem-solving test following long-term cognitive test exposure. *Royal Society Open Science*, *8*(10), 210656. https://doi.org/10.1098/rsos.210656

Rosenberger, K., Simmler, M., Langbein, J., Nawroth, C., & Keil, N. (2022). Responsiveness of domesticated goats towards various stressors following long-term cognitive test exposure. *PeerJ*, *10*, e12893. https://doi.org/10.7717/peerj.12893

Rybarczyk, P., Koba, Y., Rushen, J., Tanida, H., & de Passillé, A. M. (2001). Can cows discriminate people by their faces? *Applied Animal Behaviour Science*, *74*(3), 175–189. https://doi.org/10.1016/S0168-1591(01)00162-9

Saikali, S., Meurice, P., Sauleau, P., Eliat, P.-A., Bellaud, P., Randuineau, G., Vérin, M., & Malbert, C.-H. (2010). A three-dimensional digital segmented and deformable brain atlas of the domestic pig. *Journal of Neuroscience Methods*, *192*(1), 102–109. https://doi.org/10.1016/j.jneumeth.2010.07.041

Saito, T., Nemoto, T., Nagase, Y., Kasuya, E., & Sakumoto, R. (2004). Development of a stereotaxic instrument for study of the bovine central nervous system. *Brain Research Bulletin*, *62*(5), 369–377. https://doi.org/10.1016/j.brainresbull.2003.07.010

Schuetz, A., Farmer, K., & Krueger, K. (2017). Social learning across species: Horses (Equus caballus) learn from humans by observation. *Animal Cognition*, *20*(3), 567–573. https://doi.org/10.1007/s10071-016-1060-8

Stenfelt, J., Yngvesson, J., Blokhuis, H. J., & Rørvang, M. V. (2022). Dairy cows did not rely on social learning mechanisms when solving a spatial detour task. *Frontiers in Veterinary Science*, *9*. https://doi.org/10.3389/fvets.2022.956559

Sunagawa, K., Nagamine, I., Fujino, T., Nakatsu, Y., & Hikosaka, K. (2015). Stereotaxic atlas of the goat brain for an accurate approach to the hypothalamic nuclei. *Physiology & Behavior*, *145*, 91–105. https://doi.org/10.1016/j.physbeh.2015.03.030

Uller, C., & Lewis, J. (2009). Horses (Equus caballus) select the greater of two quantities in small numerical contrasts. *Animal Cognition*, *12*(5), 733–738. https://doi.org/10.1007/s10071-009-0225-0

van der Staay, F. J., Gieling, E. T., Pinzón, N. E., Nordquist, R. E., & Ohl, F. (2012). The appetitively motivated “cognitive” holeboard: A family of complex spatial discrimination tasks for assessing learning and memory. *Neuroscience & Biobehavioral Reviews*, *36*(1), 379–403. https://doi.org/10.1016/j.neubiorev.2011.07.008

van der Staay, F. J., Schuurman, T., Hulst, M., Smits, M., Prickaerts, J., Kenis, G., & Korte, S. M. (2010). Effects of chronic stress: A comparison between tethered and loose sows. *Physiology & Behavior*, *100*(2), 154–164. https://doi.org/10.1016/j.physbeh.2010.02.020

Veissier, I. (1993). Observational learning in cattle. *Applied Animal Behaviour Science*, *35*(3), 235–243. https://doi.org/10.1016/0168-1591(93)90139-G

Veit, A., Wondrak, M., & Huber, L. (2017). Object movement re-enactment in free-ranging Kune Kune piglets. *Animal Behaviour*, *132*, 49–59. https://doi.org/10.1016/j.anbehav.2017.08.004

Visser, E. K., van Reenen, C. G., van der Werf, J. T. N., Schilder, M. B. H., Knaap, J. H., Barneveld, A., & Blokhuis, H. J. (2002). Heart rate and heart rate variability during a novel object test and a handling test in young horses. *Physiology & Behavior*, *76*(2), 289–296. https://doi.org/10.1016/S0031-9384(02)00698-4

Wondrak, M., Conzelmann, E., Veit, A., & Huber, L. (2018). Pigs (Sus scrofa domesticus) categorize pictures of human heads. *Applied Animal Behaviour Science*, *205*, 19–27. https://doi.org/10.1016/j.applanim.2018.05.009

Yum, S.-Y., Youn, K.-Y., Choi, W.-J., & Jang, G. (2018). Development of genome engineering technologies in cattle: From random to specific. *Journal of Animal Science and Biotechnology*, *9*(1), 16. https://doi.org/10.1186/s40104-018-0232-6
